# A Modified Constitutive Model for Isotropic Hyperelastic Polymeric Materials and Its Parameter Identification

**DOI:** 10.3390/polym15153172

**Published:** 2023-07-26

**Authors:** Wei Wang, Yang Liu, Zongwu Xie

**Affiliations:** State Key Laboratory of Robotics and Systems, Harbin Institute of Technology, Harbin 150001, China; wangwei1023@stu.hit.edu.cn (W.W.); xiezongwu@hit.edu.cn (Z.X.)

**Keywords:** hyperelastic, polymeric materials, constitutive model, strain energy density function, parameter identification

## Abstract

Given the importance of hyperelastic constitutive models in the design of engineering components, researchers have been developing the improved and new constitutive models in search of a more accurate and even universal performance. Here, a modified hyperelastic constitutive model based on the Yeoh model is proposed to improve its prediction performance for multiaxial deformation of hyperelastic polymeric materials while retaining the advantages of the original Yeoh model. The modified constitutive model has one more correction term than the original model. The specific form of the correction term is a composite function based on a power function represented by the principal stretches, which is derived from the corresponding residual strain energy when the Yeoh model predicts the equibiaxial mode of deformation. In addition, a parameter identification method based on the cyclic genetic-pattern search algorithm is introduced to accurately obtain the parameters of the constitutive model. By applying the modified model to the experimental datasets of various rubber or rubber-like materials (including natural unfilled or filled rubber, silicone rubber, extremely soft hydrogel and human brain cortex tissue), it is confirmed that the modified model not only possesses a significantly improved ability to predict multiaxial deformation, but also has a wider range of material applicability. Meanwhile, the advantages of the modified model over most existing models in the literatures are also demonstrated. For example, when characterizing human brain tissue, which is difficult for most existing models in the literature, the modified model has comparable predictive accuracy with the third-order Ogden model, while maintaining convexity in the corresponding deformation domain. Moreover, the effective prediction ability of the modified model for untested equi-biaxial deformation of different materials has also been confirmed using only the data of uniaxial tension and pure shear from various datasets. The effective prediction for the untested equibiaxial deformation makes it more suitable for the practice situation where the equibiaxial deformation of certain polymeric materials is unavailable. Finally, compared with other parameter identification methods, the introduced parameter identification method significantly improves the predicted accuracy of the constitutive models; meanwhile, the uniform convergence of introduced parameter identification method is also better.

## 1. Introduction

Hyperelastic polymeric materials, which possess exceptional hyperelastic properties, have been widely utilized in various fields such as medical devices [1], flexible electrodes [2], and soft robots [3]. The widespread applications of hyperelastic polymeric materials have also spurred a lot of research into characterizing their hyperelastic properties [4]. In particular, carrying out the analysis of the 3D stress–strain of complex elastic components relying on finite element analysis (FEA) has become an indispensable part in the process of product design in recent decades. However, the reliability and accuracy of results strongly depend on the performance of the constitutive model predicting the mechanical behavior of the material. Therefore, a constitutive model that can more accurately predict the mechanical behavior of material is the key to making the analytical results based on finite element analysis more realistic.

Currently, constitutive models characterizing hyperelastic properties are usually built by the strain energy density function. In light of the different ways of constructing the strain energy density function, the hyperelastic constitutive models can be divided into two overall categories: the first category encompass statistical mechanics models based on the idealized network structure of molecular chains, while the other category comprise phenomenological models based on continuum mechanics.

For statistical mechanical models, it is usually constructed based on the assumption of the network structure of molecular chains inside the polymer [5]. Although the parameters of these models usually have practical physical context, the statistical mechanical models with better representational ability often have a more complex form. So, they are not very good for providing an analytical solution, and they are not good for numerical solutions [6], as well as having a large corresponding calculation [7]. In addition, some statistical mechanical models are not well suited for dealing with some important issues such as irreversible deformation and inelastic volume expansion [8].

For phenomenological models, they are usually constructed based on the strain invariants or principal stretches. Although the parameters of these phenomenological models often do not have practical physical context, they have been widely studied and applied because of their relative advantages (including easy-to-obtain parameters, relatively high computational efficiency, no requirement for understanding the microstructure of the materials, and wider material applicability). The Mooney model is the earliest constitutive model used to represent the mechanical behavior of polymeric rubber. It has a linearized form, and it usually has better performance in the case with small deformation [9]. Taking into account the limited capabilities of the Mooney model, Rivlin extended it in the form of polynomial series to obtain a generalized polynomial model [10]. Because the polynomial model has higher-order terms of the strain invariants, it is more suitable for the case with large deformation. However, the model with higher-order terms usually requires more parameters, which easily make the model unstable. In line with the structure of polynomial model, researchers explored different orders and combinations of strain invariants, and derived many other models [11,12,13,14]. In particular, when the second strain invariant in the polynomial model is ignored, the reduced polynomial model is obtained [15]. Even though the polynomial model and reduced polynomial model can well characterize the hyperelastic properties of filled and unfilled rubber by retaining higher-order terms [16], they may have difficulties in solving numerical problems [17]. Therefore, the second-order polynomial model or the third-order reduced polynomial model is generally used. Except for some of the above constitutive models on account of the polynomial form of strain invariants, there are also some constitutive models based on the logarithm or exponential form of strain invariants. Notwithstanding, comparative studies show that these models do not have a particular advantage in characterizing hyperelastic properties [18,19]. Besides, the eigenvalues of the strain tensor are related to the principal stretches, so there are also some constitutive models constructed directly based on the principal stretches [20,21,22]. These models are usually composed of special functions related to the principal stretches, and they show good performance in characterizing the hyperelastic properties.

Although there are already many classical constitutive models to characterize the hyperelastic property, there are still continuously improved and completely new models emerging. The reason for this may be that the deficiencies of existing models still exist [23] and the importance of hyperelastic models in designing engineering components still motivates researchers to develop more general and robust constitutive models [24,25]. As presented in some reviews [7,26], the performances of these existing models are different from each other, and not all models can effectively characterize the multiaxial deformation of hyperelastic material based solely on a single set of model parameters, and most of these models are applied to a specific type of hyperelastic material. Furthermore, many models with relatively few parameters cannot reliably predict the whole range of strain and different modes of deformation. Considering that hyperelastic materials often exhibit multiaxial states of deformation in practical applications, it is unquestionable that the ability of a model to characterize multiaxial deformation should be evaluated first. However, evaluating the ability of a model to characterize multiaxial deformation often requires the simultaneous use of experimental data from three modes of homogenous deformation (including uniaxial tension, equibiaxial tension, and pure shear) to calibrate the parameters of the model. This may be prohibitively expensive and even infeasible in practice [15]. Due to limited hardware conditions or limitations in the tensile strength of the target hyperelastic material, some pure deformation mode data of materials are simply not measurable, especially for the equibiaxial tension. So, there are also some researchers evaluating the performance of existing models from another dimension. As presented in the comparative studies in [15,27], the parameters of these models were calibrated only using data from a single pure mode of deformation (such as uniaxial tension). The corresponding comparison results highlight the effective characterization ability of Yeoh model [28] for untested deformation modes (exceeding the well-known Ogden model [21]). Although some studies believe that it may not be reliable for a model only calibrated through the data of a single mode to describe the multiaxial mode of deformation [6,29], as an auxiliary evaluation method, it is also being pursued by researchers [18,30,31,32]. Especially in [3], researchers refer to the model’s ability to effectively predict the untested mode of deformation as the property of deformation-mode independency. As described above, this property is particularly useful for real-world engineering applications where the available experimental data are limited [32].

Considering the pursuit of a universal and robust hyperelastic constitutive model, this study aims to improve the Yeoh model to compensate for its shortcoming in performance and applicability. The idea of focusing on improving the Yeoh model is not arbitrary, but rather based on its unique advantages in characterizing untested modes of deformation as explained above. The reason why we want to take advantage of this advantage is because we have indeed encountered the situation in practice where the equibiaxial deformation mode of a used polymer material is not measurable. With the widespread application of different soft polymer materials in engineering design, we believe that the probability of this situation occurring will be higher. So, the actual demand also drives us to carry out this work. Furthermore, it is well known that the Yeoh model will underestimate the equibiaxial mode of deformation when characterizing multiaxial state of stress; hence, it should be further improved in order to have more accurate characterization results. Hitherto, there are several sporadic improved Yeoh models. Earlier, Yeoh proposed to improve the fitting accuracy of the Yeoh model to the data from simple shear by adding an additional exponential term related to the first principal invariant [33]. The comparative studies confirmed that the improved model has a poorer performance for predicting the biaxial behavior of natural rubber (using Treloar’s data), and the comprehensive performance of the improved model is not as good as the original Yeoh model [34]. This may be because the improved model is still not related to the second principal invariant or the added additional exponential item is inappropriate. Relevant study has confirmed that the model containing the second principal invariant is important for improving the prediction accuracy of the model, especially for the equibiaxial deformation [35]. Based on this, a recently improved Yeoh model adds the square root of the second principal invariant as a correction term to the original Yeoh model [30]. The research results confirm that the modified model has significantly improved predictive performance for equibiaxial tension, but the entire modification process is based on uniaxial data, which may lead to insufficient improvement in the model’s actual predictive ability for multiaxial deformations. In addition, it has not yet imposed effective constraints on the parameters of the modified model to ultimately make the modified model convex and stable. In addition to these two modified Yeoh models mentioned above, Hohenberger et al. also replaced all the determined orders in the Yeoh model with undetermined coefficients to expand it into a generalized Yeoh model, so as to describe the low and high strain nonlinearity of highly filled high damping rubber [36]. This generalized Yeoh model has been proven to have improved prediction accuracy for uniaxial tension and compression, but its characterization accuracy for multiaxial deformation has not been determined. To sum up, there is still a certain distance between the current modified Yeoh models and the universal and robust model; so, it is still meaningful to improve the Yeoh model again.

In this study, we use a thoughtful correction term to modify the Yeoh model, so as to improve its ability for characterizing multiaxial deformations. This correction term is derived from the corresponding residual strain energy when the Yeoh model predicts the equibiaxial mode of deformation, and its specific form is a composite function based on a power function represented by the principal stretches. The strain energy density function of the modified model is represented by the first strain invariant and the principal stretches, and it contains only five parameters to be identified. In the case of specific parameters, the model can be degraded to the neo-Hookean model, the Mooney–Rivlin model, the Yeoh model, and the Biderman model. Therefore, the modified model can also be regarded as the parent model of these four classical models. In addition, we also introduce a special parameter identification method based on the cyclic genetic-pattern search algorithm in order to improve the predicted accuracy of constitutive models. For demonstrating the modified model’s robustness and generality, the modified model is applied to six different types of hyperelastic materials. During this process, in addition to comparing the performance of the modified constitutive model with some landmark constitutive models in the literatures, the stability and convexity of the modified constitutive model is also verified. Finally, the validity of the introduced parameter identification method is also confirmed by comparing it with four existing parameter identification methods.

## 2. Materials and Methods

### 2.1. Experimental Data

Experimental data involving simple modes of deformation like uniaxial tension (UT), equibiaxial tension (ET) and pure shear (PS) form the basis for parameter identification of the constitutive model. And, it is a common practice to contrast the predicted results of model and experimental data for examining the performance of the model in different ranges of deformation and different modes of deformation. Here, considering the widespread use of the experimental dataset about natural unfilled vulcanized rubber obtained by Treloar [37], we take the lead in using it to obtain the correction term of the Yeoh model. Then, we will analyze the performance of the modified model based on this dataset. Given that Treloar’s dataset contains a large range of deformation, the dataset is divided into three ranges of deformation for providing a more detailed evaluation of the model’s ability to describe different ranges of deformation [26]. That is, the experimental data in each mode of deformation (UT, ET and PS) will be divided into three ranges of deformation based on the principal stretch, namely, small deformation ($1<\lambda <\lambda_{\max}/3$), medium deformation ($1<\lambda <2\lambda_{\max}/3$) and large deformation ($1<\lambda <\lambda_{\max}/3$). $\lambda_{\max}$, respectively, represents the maximum principal stretch of UT, ET and PS. Although the cutoff ranges of data in different deformation ranges under the same mode of deformation are different, they all belong to the complete stress–strain curve under the corresponding deformation mode. In addition, these data with different ranges of deformation are smoothed and homogenized to obtain more data points and minimize measurement noise as much as possible, thereby obtaining more accurate results of parameter estimation. The specific data points can be found in Table S1.

Furthermore, in order to fully verify the characterization ability of the modified model for the multiaxial deformation of different materials, we also considered experimental datasets from another five different types of rubber-like materials (including isoprene vulcanized rubber [38], unfilled silicone rubber [17], poly-acrylamide hydrogel [39], carbon-black-filled styrene butadiene rubber [40] and human brain cortex tissue [41]), which have been also used in other literatures [24,25,42]. Except for the dataset of human brain cortex tissue, the other four datasets all contain data from three deformation modes of UT, ET and PS (see Tables S2–S6 in the Supporting Information for details). Overall, we used a total of six experimental datasets, which is extremely rare in other studies. Based on the six datasets, the comprehensive applicability of the modified model proposed in this study to different types of rubber-like materials will be fully demonstrated.

In addition to applying the proposed model to more material datasets, comparing the modified model proposed in this study with some commonly used or newly established models in existing studies is also beneficial for visually demonstrating the capabilities of the proposed model. To this effect, we selected a total of eight constitutive models that exist in the literature for comparison. The selection of these constitutive models for comparison is not arbitrary. Firstly, considering that this study is an improvement on the Yeoh model [28], the Yeoh model and the other three improved Yeoh models (including the modified Yeoh model proposed by Yeoh [33], generalized Yeoh model proposed by Hohenberger [36] and Melly model proposed by Melly [30]) are first added to the comparison list to verify that the improvement in this study is more effective. Secondly, in order to investigate whether the improved model in this study also holds an advantage in the current existing catalog of constitutive models, the powerful and universal third-order Ogden model [21] and the Alexander model with the best performances in the category of phenomenological models [26,43] have been added to the comparison list. Finally, under the persuasion of the reviewer, a model (we call it the Anssari–Benam model) recently proposed by him in [24] and a model (we call it the modified Anssari–Benam model) obtained by improving the former based on our correction term in our study have also been added to our comparison list. We believe that by comparing our proposed modified model with the aforementioned models on different material datasets, the performance and even advantages of our modified model will be presented more intuitively.

### 2.2. Preliminaries

In view of the assumptions of isotropy and incompressibility for hyperelastic polymeric materials, the constitutive equation of the isotropic and incompressible hyperelastic polymeric materials can be expressed as follows:

(1) $$\sigma =-pI-2\frac{\partial W}{\partial I_{2}}B^{-1}+2\frac{\partial W}{\partial I_{1}}B$$

where

(2) $$\begin{array}{l} I_{1}=trB=\lambda_{1}^{2}+\lambda_{2}^{2}+\lambda_{3}^{2}\\ I_{2}=\frac{1}{2}\left[{\left(trB\right)}^{2}-trB^{2}\right]=\lambda_{1}^{2}\lambda_{2}^{2}+\lambda_{2}^{2}\lambda_{3}^{2}+\lambda_{3}^{2}\lambda_{1}^{2} \end{array}$$

In the above equations, $I_{1}$ and $I_{2}$ are the first and second invariants of the left Cauchy–Green strain tensor B, respectively; $\lambda_{1}$, $\lambda_{2}$ and $\lambda_{3}$ are principal stretches; p is an undetermined pressure which is independent of deformation; the strain energy density function W is only a function of the first and second invariants corresponding to tensor-type arguments.

For isotropic hyperelastic polymeric materials, the principal axis of the Cauchy stress tensor σ, left Cauchy–Green strain tensor B and its inverse $B^{-1}$ are the same, so the component form of the constitutive equation of the hyperelastic polymeric materials in its two principal directions can be expressed as follows:

(3) $$\sigma_{\alpha }=-p-2\frac{\partial W}{\partial I_{2}}•\frac{1}{\lambda_{\alpha }^{2}}+2\frac{\partial W}{\partial I_{1}}•\lambda_{\alpha }^{2}$$

(4) $$\sigma_{\beta }=-p-2\frac{\partial W}{\partial I_{2}}•\frac{1}{\lambda_{\beta }^{2}}+2\frac{\partial W}{\partial I_{1}}•\lambda_{\beta }^{2}$$

where $\lambda_{\alpha }$ and $\lambda_{\beta }$ are, respectively, the principal stretch of main direction α and β.

The strain energy density function also has the following relationships to the principal stretches:

(5) $$\frac{\partial W}{\partial \lambda_{\alpha }}=2\lambda_{\alpha }\frac{\partial W}{\partial I_{1}}-\frac{2}{\lambda_{\alpha }^{3}}\frac{\partial W}{\partial I_{2}}$$

(6) $$\frac{\partial W}{\partial \lambda_{\beta }}=2\lambda_{\beta }\frac{\partial W}{\partial I_{1}}-\frac{2}{\lambda_{\beta }^{3}}\frac{\partial W}{\partial I_{2}}$$

According to Equations (3)–(6), the following expression can be obtained by eliminating p:

(7) $$\sigma_{\alpha }-\sigma_{\beta }=\lambda_{\bar{\alpha }}\frac{\partial W}{\partial \lambda_{\bar{\alpha }}}-\lambda_{\bar{\beta }}\frac{\partial W}{\partial \lambda_{\bar{\beta }}}$$

where $\bar{\alpha }$ and $\bar{\beta }$ are not summation indices; so, do not sum them up.

### 2.3. The Modified Strain Energy Density Function

**Figure 1:**
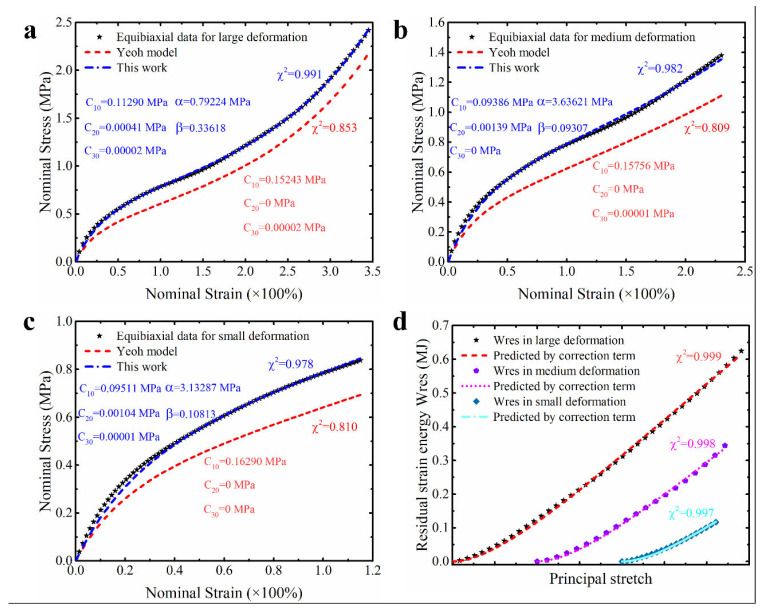
The characterization ability of Yeoh model for equibiaxial deformation mode under different ranges of deformation: (**a**) large deformation; (**b**) medium deformation; (**c**) small deformation; (**d**) residual strain energy under different ranges of deformation. The model parameters here are obtained by using experimental data of natural unfilled vulcanized rubber from uniaxial tension, equibiaxial tension and pure shear at the same time.

As stated in the introduction, the third-order polynomial based on the first strain invariant (Yeoh model) not only has a simple form, but can also reproduce the inverse S-shape of the stress–strain curve of the hyperelastic polymeric materials under different modes of deformation [28] and it also has the ability to effectively predict untested deformation modes of natural unfilled vulcanized rubber [15,27]. However, it is always underestimating the equibiaxial mode of deformation (as shown in Figure 1). This underestimation will greatly reduce its ability to predict multiaxial deformation, thus hindering its application in actual engineering [30]. Some studies have shown that the second strain invariant is also important and making the model include the second strain invariant can achieve higher precision for predicting the multiaxial stress state [35]. For example, because the Biderman model has an additional term (including the second strain invariant) compared to the Yeoh model, the Biderman model has better prediction accuracy for the equibiaxial deformation of natural unfilled vulcanized rubber [44]. Nonetheless, the Biderman model may incorrectly predict the deformation mode without the corresponding calibrated data [30]. Hence, the benefits obtained by simply adding the second strain invariant to the Yeoh model are limited.

In order to more effectively improve the characterization ability of the Yeoh model for the equibiaxial mode of deformation, the corresponding residual strain energy is calculated (as shown in Figure 1d). Based on the analysis of residual strain energy, we found that a composite function based on a power function represented by the principal stretches can perfectly predict the residual strain energy (as shown in Figure 1d). Considering the function predicting the residual strain energy as a correction term, a modified strain energy density function is proposed in the following form:

(8) $$W=C_{10}\left(I_{1}-3\right)+C_{20}{\left(I_{1}-3\right)}^{2}+C_{30}{\left(I_{1}-3\right)}^{3}+\frac{\alpha }{\beta }\left[{\left(\lambda_{1}\lambda_{2}\right)}^{\beta }+{\left(\lambda_{2}\lambda_{3}\right)}^{\beta }+{\left(\lambda_{1}\lambda_{3}\right)}^{\beta }-3\right]$$

The correction term of modified strain energy density function can be understood as a generalization of the first-order form about the second strain invariant. When β=2, the correction term degenerates to the first-order form about the second strain invariant. In this point, the modified model becomes the Biderman model. In addition, the modified strain energy function can also be reduced to the neo-Hookean model ($C_{20}=C_{30}=\alpha =0\;\mathrm{MPa}$), Yeoh model (α=0 MPa) and Mooney–Rivlin model ($C_{20}=C_{30}=0\;\mathrm{MPa},\beta =2$) under the condition of a specific coefficient. Therefore, the modified model can also be regarded as the parent model of these four classical models. Furthermore, reducing the higher-order terms of third-order polynomials can also make Equation (8) turn into other forms, whose performance will be explained later.

#### 2.3.1. Constitutive Restrictions of the Proposed Strain Energy Density Function

Considering the material objectivity and the consistency between the general theory of isotropic elasticity and its classical linear theory, six general postulates for the strain energy density function are proposed by Ogden [45]. Based on these postulates, the modified strain energy density function should satisfy the following relationships:

(9) $$\frac{\partial^{2}W\left(\lambda_{1}=1,\lambda_{2}=1\right)}{\partial \lambda_{i}{}^{2}}=8C_{10}+2\alpha \beta >0$$

(10) $$\begin{array}{l} \det\left[H_{ij}\right]>0,\\ H_{ij}=\frac{\partial^{2}W\left(\lambda_{1}=1,\lambda_{2}=1\right)}{\partial \lambda_{i}\partial \lambda_{j}},i,j=1,2 \end{array}$$

In view of the above formulas, and in order to make the model consistent with its four degraded models, here, the following constraint for the coefficient of strain energy density function is kept:

(11) $$C_{10}>0;\;4C_{10}+\alpha \beta >0$$

Furthermore, some researchers gradually regard the poly-convexity as a fundamental mathematical constraint to guarantee the existence of a solution for the constitutive model in the boundary-value problem [46]. Here, the stricter condition of convexity, which requires the Hessian matrix of Equation (8) to be positive definite [42], is adopted. According to the components in the Hessian matrix (see Equation (S1) in the supporting information), ***H*** is symmetric. Therefore, the conditions for making ***H*** positive definite are as follows:

(12) $$\{\begin{array}{c} \begin{array}{cc} \frac{\partial^{2}W}{\partial \lambda_{1}{}^{2}}>0, & \frac{\partial^{2}W}{\partial \lambda_{2}{}^{2}}>0 \end{array}\\ \frac{\partial^{2}W}{\partial \lambda_{1}{}^{2}}\cdot \frac{\partial^{2}W}{\partial \lambda_{2}{}^{2}}-\frac{\partial^{2}W}{\partial \lambda_{1}\partial \lambda_{2}}\cdot \frac{\partial^{2}W}{\partial \lambda_{2}\partial \lambda_{1}}>0 \end{array}$$

Hence, as long as the inequality (12) is satisfied, the constitutive model with calibrated parameters will possess the poly-convexity.

#### 2.3.2. The Stability Criterion of the Proposed Strain Energy Density Function

A stable constitutive model obeying the laws of thermodynamics is a prerequisite for subsequent finite element analysis. An unstable strain energy function will adversely affect the nonlinear numerical algorithms in the finite element codes [47]. If the obtained constitutive model is unstable, it is necessary to re-determine the parameters of the model or even replace other models.

According to the Drucker stability criterion used in the commercial finite element software [47], the relation between changes in the principal stress and changes in the principal strain can be described by the following matrix equation:

(13) $$\left[\begin{array}{c} d\sigma_{1}\\ d\sigma_{2} \end{array}\right]=\left[\begin{array}{cc} D_{11} & D_{12}\\ D_{21} & D_{22} \end{array}\right]\left[\begin{array}{c} d\varepsilon_{1}\\ d\varepsilon_{2} \end{array}\right]$$

If the hyperelastic constitutive model is stable, it is only required that the coefficient matrix (***D***) of the above matrix equation is positive definite. The components of the coefficient matrix (***D***) are as shown in Equation (S2) in the supporting information.

### 2.4. Analytical Stress Formulations for Standard Tests

The mechanical behavior of hyperelastic polymeric materials can be determined by standard tests involving simple modes of deformation such as uniaxial tension (UT), equibiaxial tension (ET) and pure shear (PS). The relationships between nominal stress and principal stretches under these simple modes of deformation can be derived from Equations (7) and (8) as follows:

(14) $$T_{\mathrm{UT}}=\frac{1}{\lambda }\cdot \left[C_{10}+2C_{20}\cdot \left(I_{1}-3\right)+3C_{30}\cdot {\left(I_{1}-3\right)}^{2}\right]\cdot \left(2\lambda^{2}-\frac{2}{\lambda }\right)+\frac{1}{\lambda }\cdot \left[\alpha \cdot \left(\lambda^{\frac{\beta }{2}}-\lambda^{-\beta }\right)\right]$$

(15) $$T_{\mathrm{ET}}=\frac{1}{\lambda }\cdot \left[C_{10}+2C_{20}\cdot \left(I_{1}-3\right)+3C_{30}\cdot {\left(I_{1}-3\right)}^{2}\right]\cdot \left(2\lambda^{2}-\frac{2}{\lambda^{4}}\right)+\frac{1}{\lambda }\cdot \left[\alpha \cdot \left(\lambda^{2\beta }-\lambda^{-\beta }\right)\right]$$

(16) $$T_{\mathrm{PS}}=\frac{1}{\lambda }\cdot \left[C_{10}+2C_{20}\cdot \left(I_{1}-3\right)+3C_{30}\cdot {\left(I_{1}-3\right)}^{2}\right]\cdot \left(2\lambda^{2}-\frac{2}{\lambda^{2}}\right)+\frac{1}{\lambda }\cdot \left[\alpha \cdot \left(\lambda^{\beta }-\lambda^{-\beta }\right)\right]$$

### 2.5. Parameter Identification of Model

Identifying the parameters of the constitutive model is an important step during the characterization of the hyperelasticity. Although some optimization algorithms have been used to identify the parameters of constitutive models, these used methods still have their own problems. For example, the damped least squares algorithm based on nonlinear least squares [48] and the sequential quadratic programming algorithm based on multi-objective optimization strategy [49] are all affected by initial values. The genetic algorithm using multi-objective optimization strategy has deficiencies in terms of local search capability [50]. Although the hybrid optimization algorithm based on the pattern search and damped least squares can improve the local search capability of the single-optimization algorithm, its global search capability is not as strong as the genetic algorithm [51]. In order to obtain more accurate parameters of the model, the sensitivity of the identification method to the initial values and its search capability need to be considered.

**Figure 2:**
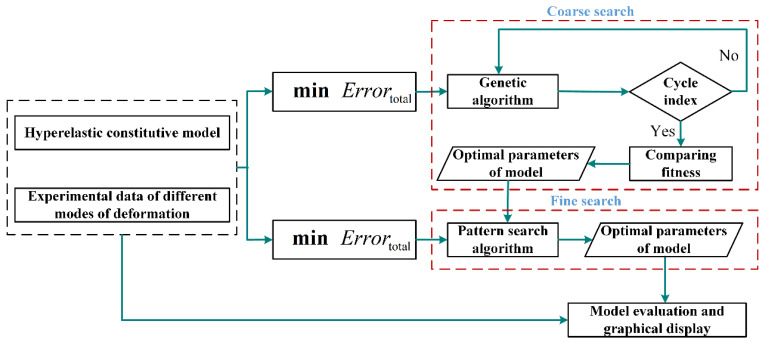
The framework of parameter identification of constitutive model based on cyclic genetic-pattern search algorithm.

On these grounds, this study introduces a parameter identification method of the constitutive model based on the cyclic genetic-pattern search algorithm, and its framework is shown in Figure 2. In order to contain the experimental data with different modes of deformation during the process of parameter identification as much as possible and effectively weigh the importance of different modes of deformation, a weighted multi-objective optimization strategy is adopted. To better measure the error between the predicted results of the model and experimental data, the objective function is defined as shown in Equation (17). For obtaining the global optimal solution during solving the optimization problem as much as possible, the hybrid optimization algorithm based on genetic-pattern search is employed. The method utilizes the genetic algorithm for global coarse search and utilizes the pattern search algorithm for local fine search. The combination of coarse search and fine search makes the result closer to the ideal optimal value. Moreover, for further improving the accuracy of the model and reducing the uncertainty caused by the random search, the cyclic item based on the genetic algorithm is added to the process of identification. Before the cyclic condition terminates, the genetic algorithm independently operates multiple times, and its corresponding results are also stored in sequence. Afterwards, the optimal set of parameters is extracted from the results of multiple runs as the initial values for the subsequent pattern search algorithm, thereby completing local fine search. The parameters obtained by the pattern search algorithm are the final optimized parameters, which will be directly used to draw the prediction curve and relative error curve of the model, so as to intuitively evaluate the performance of the model. In order to effectively evaluate the performance of constitutive model, the goodness of fit described in Equation (18) will be used to measure the capability of the model to characterize different modes of deformation, and the total error described in Equation (17) will be used to measure the overall predicted accuracy of the model. The smaller the total error is, the higher the overall predicted accuracy of the model is. And the closer the goodness of fit is to 1, the better the model characterizes the corresponding modes of deformation.

(17) $$min:Error_{\mathrm{total}}=\sum_{p=1}^{3}w_{p}•\left(\sqrt{\sum_{i=1}^{m_{p}}{\left(T_{pi}^{\mathrm{model}}-T_{pi}^{\mathrm{experiment}}\right)}^{2}}/\sqrt{\sum_{i=1}^{m_{p}}{\left(T_{pi}^{\mathrm{experiment}}\right)}^{2}}\right)$$

(18) $$\chi_{p}^{2}=1-\sqrt{\sum_{i=1}^{m_{p}}{\left(T_{pi}^{\mathrm{model}}-T_{pi}^{\mathrm{experiment}}\right)}^{2}}/\sqrt{\sum_{i=1}^{m_{p}}{\left(T_{pi}^{\mathrm{experiment}}\right)}^{2}}$$

where *p* represents the simple mode of deformation such as uniaxial tension (*p* = 1), equibiaxial tension (*p* = 2) and pure shear (*p* = 3), respectively; $m_{p}$ is the number of the experiment data for the *p*th mode of deformation; $Error_{\mathrm{total}}$ corresponds to the total error between the predicted results of the model and experimental data; $T_{pi}^{\mathrm{model}}$ and $T_{pi}^{\mathrm{experiment}}$ are the prediction stress of the model and experimental stress under *p*th mode of deformation, respectively; and $w_{p}$ is the weight for different modes of deformation, which satisfies $w_{1}+w_{2}+w_{3}=1$. Specifically, when experimental data from three deformation modes are used for identifying the parameters of the model simultaneously, the corresponding weight $w_{p}=1/3$; when experimental data with only two modes of deformation are used for parameter identification simultaneously, the corresponding weight $w_{p}=1/2$ (the weight of the unused mode of deformation is 0); when experimental data with only one mode of deformation is used, the corresponding weight $w_{p}=1$ (the weight of the other two unused modes of deformation is 0). $\chi_{p}^{2}$ is the goodness of fit for the *p*th mode of deformation.

## 3. Results and Discussion

### 3.1. Parameter Identification and Validation of Model

#### 3.1.1. Predictive Ability of the Modified Model to the Single Deformation Mode

**Figure 3:**
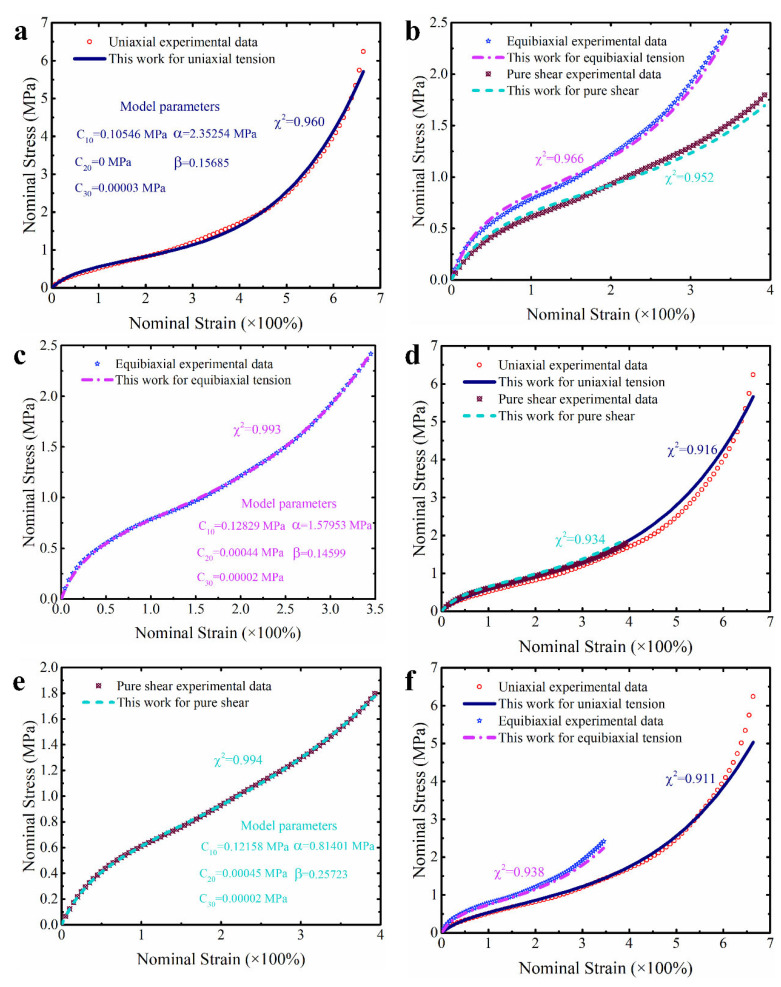
The predictive effect of the modified model on the single deformation mode. (**a**,**b**) The predictive effect of the modified model calibrated based on the single set of experimental data of uniaxial tension. (**c**,**d**) The predictive effect of the modified model calibrated based on the single set of experimental data of equibiaxial tension. (**e**,**f**) The predictive effect of the modified model calibrated based on the single set of experimental data of pure shear.

The ability of a constitutive model to accurately predict a single mode of deformation is its foundation to effectively predict multiaxial modes of deformation. In view of this, the single set of deformation data (UT, ET or PS) of natural vulcanized rubber from a large deformation range are first used to identify the parameters of the proposed modified model, respectively. Figure 3 presents the predictive effect of the modified model calibrated based on the single deformation data on the corresponding single deformation mode. It is clear that the independent predictive effect of the modified model is excellent for the two deformation modes of equibiaxial tension and pure shear, and their goodness of fit reaches 0.99 (see Figure 3c,e). Although its independent prediction effect for the deformation mode of uniaxial tension is slightly inferior, its prediction error for uniaxial tension does not exceed 4% (see Figure 3a). Based on this, we can conclude that this modified model has the ability to independently predict three deformation modes of uniaxial tension, equibiaxial tension and pure shear, which lays the foundation for its ability to effectively predict the multiaxial deformation of hyperelastic material. Here, the modified models, respectively calibrated based on the tested data from uniaxial tension, equibiaxial and pure shear, are also used to predict the other two untested deformation modes. As shown in Figure 3b,d,f, the modified model calibrated based on single set of tested data can also reasonably predict the other two untested deformation modes. As we explained in the introduction, although researchers have mixed opinions on the method to calibrate the parameters of the model based solely on data from a single deformation mode, the ability to effectively predict untested deformation modes is extremely helpful for characterizing an unmeasurable deformation mode of certain polymeric materials in practice. Anyway, compared with the results of other studies [30], the results here indicate that the additional correction term we added enhances the model’s ability to characterize untested deformation modes. This point will also continue to be discussed in a more reliable manner in the subsequent content.

#### 3.1.2. Predictive Ability of the Modified Model to the Multiaxial Deformation Mode

For verifying the prediction ability of the modified model for the multiaxial deformation mode, the data of multiple deformation modes (UT, ET and PS) from the same material dataset are simultaneously used to identify the parameters of the modified model. The following will present the predictive effects of the modified constitutive model on the multi axial deformation state of different materials in sequence. And these results are also compared with the results of other existing models (as mentioned in Section 2.1).

##### Natural Vulcanized Rubber

As mentioned earlier, the widespread use of Treloar’s experimental dataset on the natural vulcanized rubber has made it a barometer for evaluating the ability of constitutive models to characterize hyperelastic materials. Using this dataset to calibrate the modified model proposed in this study will facilitate an intuitive comparison with some models in the literature (including those models that are not on our comparison list). Table 1 summarizes the quantitative results of different constitutive models for characterizing the hyperelastic property of the natural vulcanized rubber. It can be seen from the corresponding total error that the proposed constitutive model is not only more capable in characterizing the states of multiaxial stress than the Yeoh model and the corresponding improved models (such as the modified Yeoh model, generalized Yeoh model and Melly model), but also performs better than the Anssari–Benam model newly proposed by a reviewer. And its total predicted error for different ranges of deformation is all less than 3%. In addition, the performance of the model proposed in this study also outperforms the performance of the modified Anssari–Benam model. The modified Anssari–Benam model is made by us under the persuasion of a reviewer, which is an improvement on the original Anssari–Benam model based on our correction term. Although the modified Anssari–Benam model showed significant improvement in characterizing medium and small deformations compared to the original model, it showed significant bias in characterizing uniaxial tension under large deformation. Compared with the excellent third-order Ogden model and Alexander model, our model outperforms the third-order Ogden model in characterizing large deformation, but is slightly weaker than the Alexander model. The reason why it is weaker than the Alexander model is because the goodness of fit of the proposed model for UT under large deformation is slightly lower than the Alexander model. Based on the fact that industrial rubber materials typically experience a strain range of 0–100% [32,36,52], we believe that this weakness does not affect the use of the modified model proposed in this study. Moreover, the quantitative data in Table 1 also prove that the modified model’s ability to characterize medium to small deformations is comparable to the third-order Ogden model. From a quantitative point of view, the total prediction errors of the two models are within the range of 1–1.5%, and the corresponding difference between their goodness of fit under different modes of deformation is not more than 0.5%. The overall performance of the Alexander model in characterizing medium deformation is slightly weaker than that of our proposed model.

**Figure 4:**
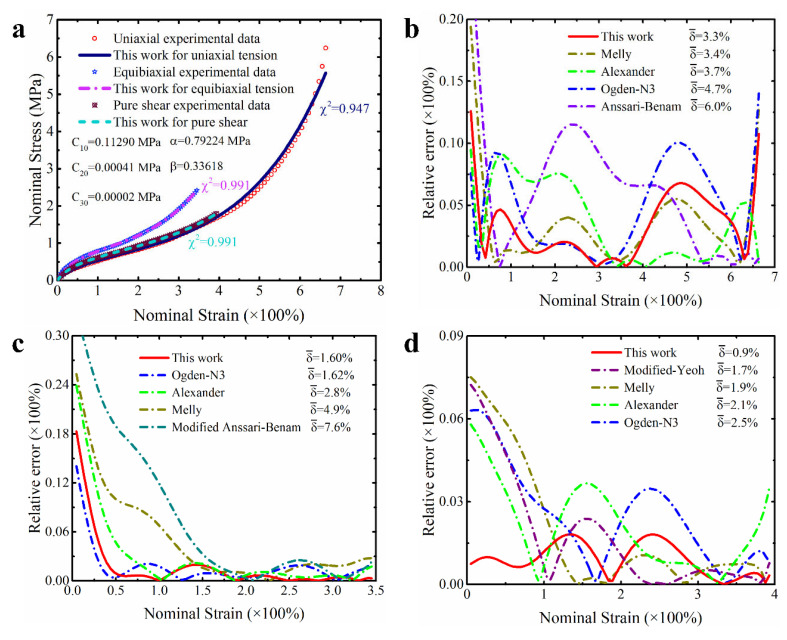
The prediction performance and corresponding prediction error curves of modified constitutive model in large deformation ranges for different deformation modes. (**a**) Prediction curves for different deformation modes; (**b**) relative error of different models in predicting uniaxial deformation mode; (**c**) relative error of different models in predicting equibiaxial deformation mode; (**d**) relative error of different models in predicting the deformation mode of pure shear. Note: Only the top five models are presented in Figure 4b–d. $\bar{\delta }$ means the average relative error.

**Figure 5:**
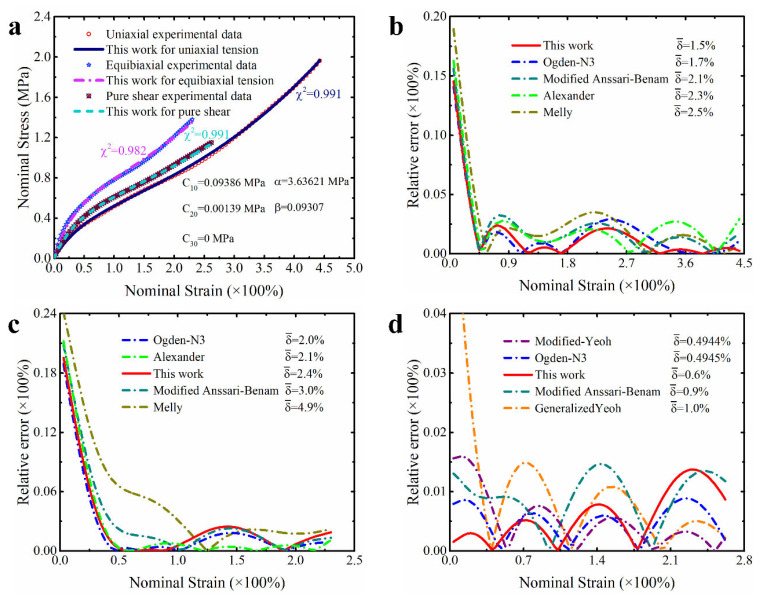
The prediction performance and corresponding prediction error curves of modified constitutive model in medium deformation ranges for different deformation modes. (**a**) Prediction curves for different deformation modes; (**b**) relative error of different models in predicting uniaxial deformation mode; (**c**) relative error of different models in predicting equibiaxial deformation mode; (**d**) relative error of different models in predicting the deformation mode of pure shear. Note: Only the top five models are presented in Figure 5b–d. $\bar{\delta }$ means the average relative error.

**Figure 6:**
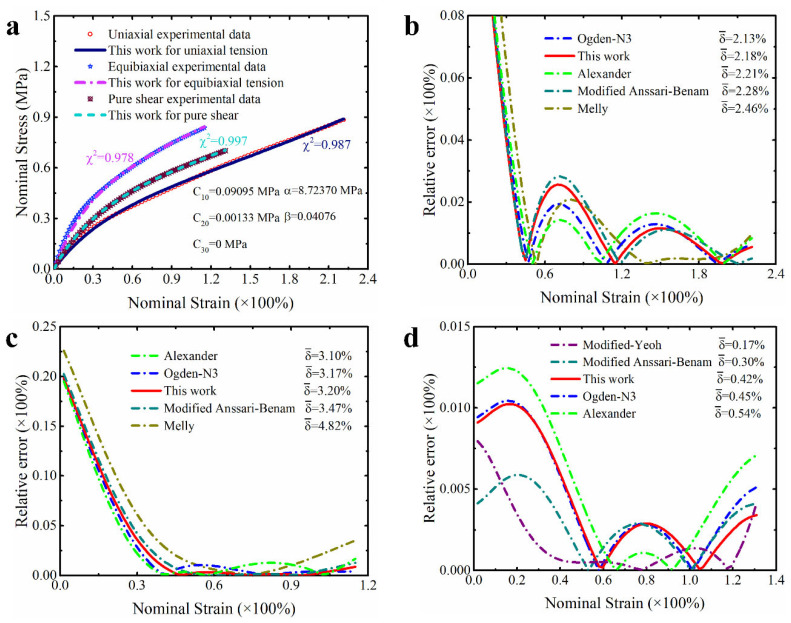
The prediction performance and corresponding prediction error curves of modified constitutive model in small deformation ranges for different deformation modes. (**a**) Prediction curves for different deformation modes; (**b**) relative error of different models in predicting uniaxial deformation mode; (**c**) relative error of different models in predicting equibiaxial deformation mode; (**d**) relative error of different models in predicting the deformation mode of pure shear. Note: Only the top five models are presented in Figure 6b–d. $\bar{\delta }$ means the average relative error.

In order to make these results more visible and clear, Figure 4, Figure 5 and Figure 6 present the prediction curves and corresponding prediction error curves of the modified constitutive model within different deformation ranges for different deformation modes, respectively. Obviously, these qualitative curves also reflect the good predicted capability of the modified model. Especially from the corresponding relative error curves, it can be seen that the relative error of the proposed model for predicting different deformation modes under different deformation ranges is comparable to the third-order Ogden model and Alexander model (their average relative errors are less than 5%), and all the average relative errors of our proposed model do not exceed 3.5%. Even for predicting the uniaxial tension under large deformation, the average relative error of the modified model proposed in this study is only 3.3%.

Further comparing the predicted results of the modified constitutive model with the Yeoh model, modified Yeoh model and generalized Yeoh model, it can be found that the overall predicted accuracy of the proposed model is several times higher than theirs in all ranges of deformation. In particular, the predicted accuracy on the equibiaxial mode of deformation is improved most obviously (as shown in Figure 1a–c). Continuing to compare with the new Melly model (it uses $\sqrt{I_{2}}$ as the correction term to improve the performance of the Yeoh model), the modified constitutive model proposed in this study is better in both overall prediction accuracy and goodness of fit for different deformation modes (the relative error curves shown in Figure 4, Figure 5 and Figure 6 can also directly reflect this point). Based on these findings, we have reason to believe that the correction term expressed in the form of a power function based on the principal stretches is beneficial in improving the ability of the model to characterize the state of multiaxial stress.

Focusing on the coefficient of the proposed model in Figure 4, Figure 5 and Figure 6, it is clear that the coefficient of the third-order term of the first strain invariant is a small value in the case of large deformation, while it changes to zero in the case of medium or small deformation. This shows that the coefficient of the third-order term of the first strain invariant cannot be ignored under the condition of large deformation. It can be speculated that the coefficient should affect the capability of the proposed model to characterize large deformation. In other words, the proposed constitutive model needs five parameters when characterizing the large deformation, while it requires only four parameters when it characterizes a small or medium deformation, which makes the proposed model more simple.

##### Isoprene Vulcanized Rubber

**Figure 7:**
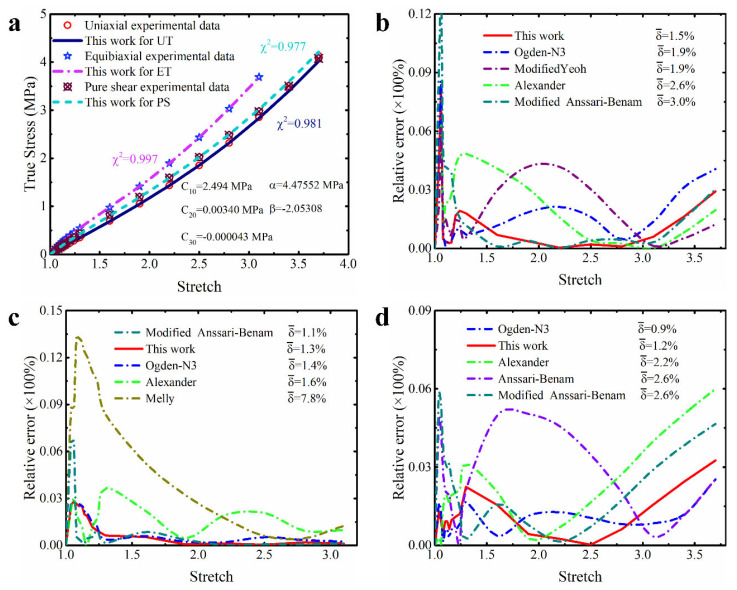
The prediction performance and corresponding prediction error curves of modified constitutive model for isoprene vulcanized rubber. (**a**) Prediction curves for different deformation modes; (**b**) relative error of different models in predicting uniaxial deformation mode; (**c**) relative error of different models in predicting equibiaxial deformation mode; (**d**) relative error of different models in predicting the deformation mode of pure shear. Note: Only the top five models are presented in Figure 7b–d. $\bar{\delta }$ means the average relative error.

The isoprene vulcanized rubber obtained by Kawabata et al. [38] and the natural vulcanized rubber obtained by Treloar [37] are similar, both of which belong to unfilled rubber. Continuing to calibrate the constitutive model based on the dataset of the isoprene vulcanized rubber will further validate the performance of the modified constitutive model proposed in this study. Table 2 summarizes some quantitative results obtained based on this dataset. Obviously, compared with other constitutive models in the table, the modified model proposed in this study achieved the lowest total prediction error (1.5%). This means that the improved model has the best comprehensive description effect on the hyperelastic properties of this material. The corresponding more intuitive prediction curves for different deformation modes are shown in Figure 7a. From the graph, it can be seen that the prediction curves of this modified model for different deformation modes almost perfectly coincide with the corresponding experimental data points. In order to compare the prediction accuracy of different models for different deformation modes in more detail, the corresponding relative error curves are shown in Figure 7b–d. In order to present the results clearly, only the relative error curves of the models with the top five prediction accuracies are shown in the corresponding figures and the average relative error of the corresponding model is marked on the legend. It can be observed that almost all the models presented in these figures have relatively low relative error, while the modified model in this study has lower relative errors for predicting different deformation modes. The vast majority of its curves are below 3%, resulting in an average relative error for predicting different deformation modes ranging from 1.2% to 1.3%, which slightly outperforms the third-order Ogden model (0.9–1.9%) and the Alexander model (1.6–2.6%).

Overall, these results once again confirm the excellent predictive performance of the modified model proposed in this study on the multiaxial deformation of unfilled rubber. Specifically, the performance of the modified model has a several-fold improvement compared to other improved Yeoh models, which once again demonstrates that the improvement of the Yeoh model based on the correction term of this study is worthwhile. In addition, the improved model outperforms the modified Anssari–Benam model, indicating that the improvement of the Yeoh model based on our correction term is superior to the improvement of the Anssari–Benam model based on the same correction term.

##### Unfilled Silicone Rubber

**Figure 8:**
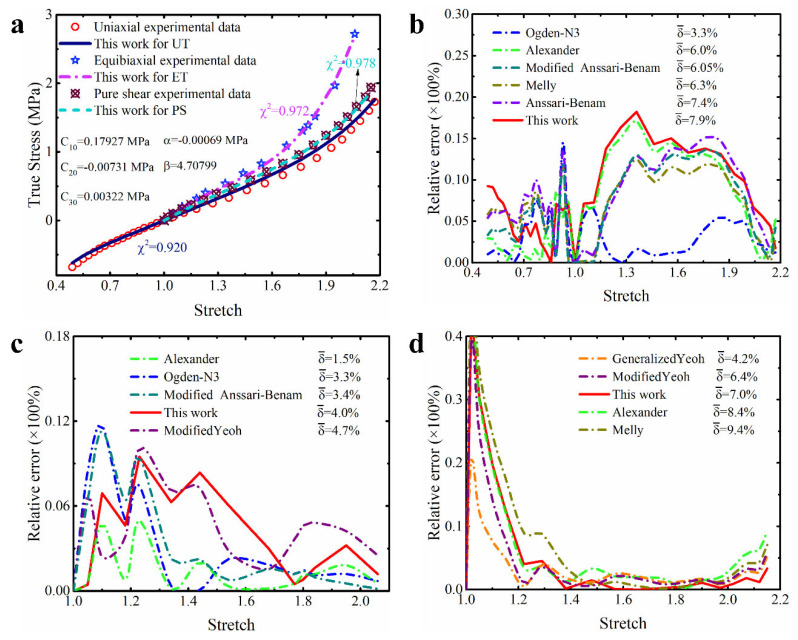
The prediction performance and corresponding prediction error curves of modified constitutive model for unfilled silicone rubber. (**a**) Prediction curves for different deformation modes; (**b**) relative error of different models in predicting uniaxial deformation mode; (**c**) relative error of different models in predicting equibiaxial deformation mode; (**d**) relative error of different models in predicting the deformation mode of pure shear. Note: Only the top five models are presented in Figure 8b–d. $\bar{\delta }$ means the average relative error.

Silicone rubber has good mechanical properties and biocompatibility [17]. Completing more accurate modeling of the mechanical properties is of great significance for its fuller utilization in the field of biomedicine. Here, the modified constitutive model proposed in this study is applied to the unfilled silicone rubber [17] to verify its usability in more precisely modeling silicone rubber. Table 3 quantitatively shows the prediction performance of different models on the hyperelastic property of unfilled silicone rubber. It can be seen from these quantitative data that although the modified constitutive model proposed in this study is not as effective as the third-order Ogden model with six undetermined parameters in predicting the multiaxial stress state of silicone rubber, it ranks second alongside the Alexander model and the modified Anssari–Benam model (both with five undetermined parameters). The reason for ranking second is that the modified model in this study slightly overestimates the uniaxial tensile deformation when characterizing the multiaxial deformations. As shown in Figure 8a, the prediction curves of the modified model for equibiaxial tension, pure shear and uniaxial compression fit well with the corresponding experimental data points, while its prediction curve for uniaxial tension is slightly higher than the experimental data points. Nonetheless, its average relative errors for predicting different deformations are no more than 8% (as shown in Figure 8b–d). And, considering that it far outperforms the Yeoh model with a total prediction error of 4.4%, and slightly surpasses these existing improved models for the Yeoh model, we still believe that the modified constitutive model proposed in this study has potential for application for unfilled silicone rubber.

##### Poly-Acrylamide Hydrogel

**Figure 9:**
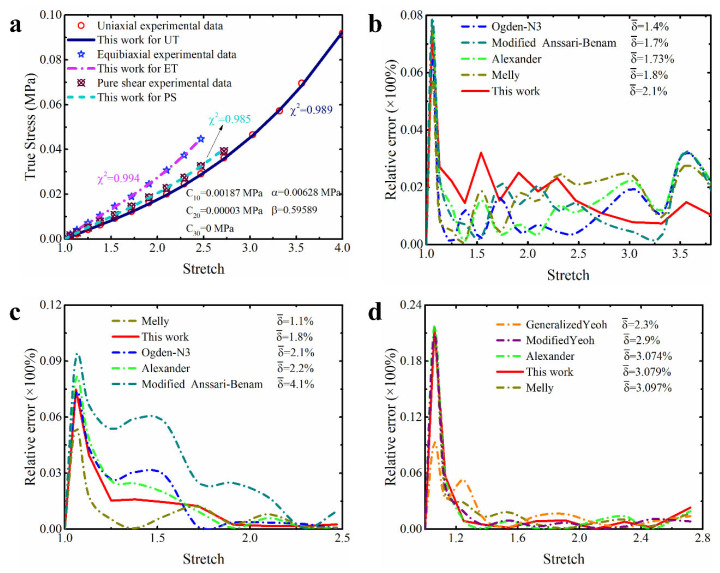
The prediction performance and corresponding prediction error curves of modified constitutive model for poly-arcylamide hydrogel. (**a**) Prediction curves for different deformation modes; (**b**) relative error of different models in predicting uniaxial deformation mode; (**c**) relative error of different models in predicting equibiaxial deformation mode; (**d**) relative error of different models in predicting the deformation mode of pure shear. Note: Only the top five models are presented in Figure 9b–d. $\bar{\delta }$ means the average relative error.

Like silicone rubber, hydrogels are also widely used in the biomedical field. But compared to silicone rubber, they undergo greater deformation due to their softness [25]. Currently, accurately characterizing their mechanical properties also has important practical significance. Here, we also apply the modified constitutive model proposed in this study to the poly-acrylamide hydrogel [39] to explore the ability of this model in describing the hyperelastic property of hydrogel. As shown in Table 4, the modified constitutive model proposed in this study has the best prediction effect on the multiaxial deformation of hydrogel. Its total prediction error for different deformation modes is only 1.1%. Figure 9 visually shows its actual prediction performance. It can be clearly seen from the figure that the prediction curves of the modified constitutive model proposed in this study for different deformations of hydrogels are in good agreement with the corresponding experimental data points, and the corresponding goodness of fit reaches 0.98. Moreover, the relative error curves of the modified model for different deformation modes are mostly below 4%, resulting in an average relative error of 1.8–3.1%. Looking carefully at the coefficients of the modified model at this time (see Figure 9a), it can be found that the coefficient of the cubic term of the first invariant is zero, which means that the modified constitutive model only needs four parameters to characterize the hydrogel. Based on the above results, we have reason to believe that the modified constitutive model also has the ability to accurately characterize the multiaxial deformation of hydrogels.

##### Carbon-Black-Filled Styrene Butadiene Rubber

**Figure 10:**
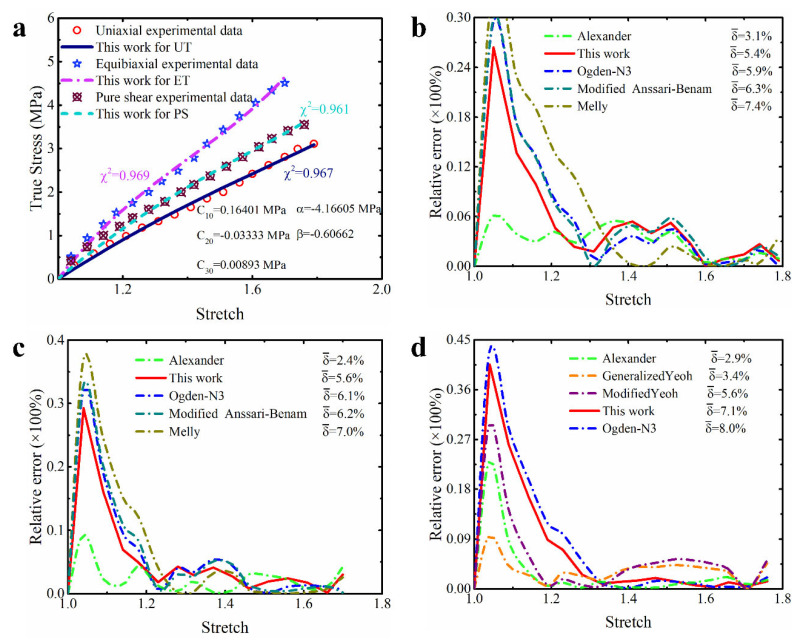
The prediction performance and corresponding prediction error curves of modified constitutive model for carbon-black-filled styrene butadiene rubber. (**a**) Prediction curves for different deformation modes; (**b**) relative error of different models in predicting uniaxial deformation mode; (**c**) relative error of different models in predicting equibiaxial deformation mode; (**d**) relative error of different models in predicting the deformation mode of pure shear. Note: Only the top five models are presented in Figure 10b–d. $\bar{\delta }$ means the average relative error.

Due to the filling of additives, the stress–strain curve of filled rubber is significantly different from that of unfilled rubber [40]. Based on this, there are few constitutive models that can properly capture the hyperelastic property of this material in the current literature [25]. If the modified constitutive model proposed in this study can also well characterize the multiaxial deformation characteristics of filled rubber, it will further confirm its adaptability to different materials. Therefore, we further apply the modified model proposed in this study to a dataset related to the carbon-black-filled styrene butadiene rubber [40]. As summarized in Table 5, the modified model proposed in this study also has good characterization ability for the multiaxial deformation of this filled rubber. Although its overall performance is not the best, it ranks behind the Alexander model and before the third-order Ogden model with a total prediction error of 3.4%. Moreover, the difference in goodness of fit between the modified model and the Alexander model for the two deformation modes of uniaxial and equibiaxial tension is no more than 1%, and the difference in goodness of fit between the two models for pure shear is only 2.2%. Furthermore, like predicting other materials, the modified model proposed in this study also outperforms other improved models of the Yeoh model in describing the multiaxial deformation of filled rubber. Figure 10 shows the intuitive prediction performance of the modified model for different deformation modes. Just as the case indicated by the aforementioned quantitative indicators, the prediction curves of the modified model for different deformation modes are in good agreement with the corresponding experimental data points (see Figure 10a) and the corresponding relative error is in a relatively low position (see Figure 10b–d). Although it can be seen from Figure 10d that the average relative error of the modified constitutive model on pure shear (7.1%) is slightly higher than that of the generalized Yeoh model and modified Yeoh model, most of the zones of its relative error curve are below 6%. Moreover, from the perspective of characterizing multiaxis deformations, there are trade-offs between the predictive performance of the model for different deformation modes during calibrating the parameters of the model, so as to optimize the overall characterization ability of the model. Based on this, the slight advantage presented by the modified Yeoh model and generalized Yeoh model in characterizing pure shear do not make them possess better overall prediction accuracy, while the result is that the modified model in this study is better than them in overall prediction accuracy. The prediction accuracy of the modified model proposed in this study for different deformation modes is relatively balanced, and each goodness of fit reaches 0.96.

##### Human Brain Cortex Tissue

**Figure 11:**
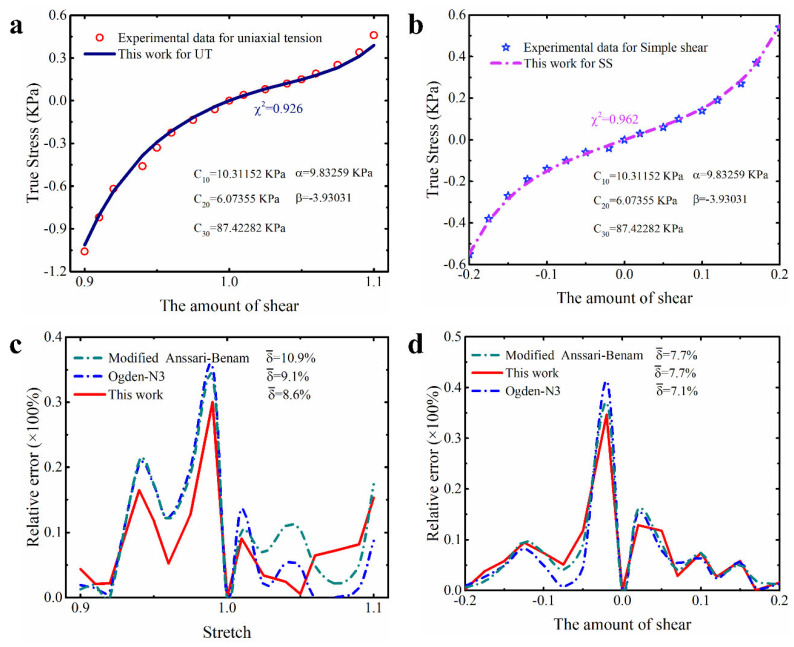
The prediction performance and corresponding prediction error curves of modified constitutive model for human brain cortex tissue. (**a**) Prediction curves for different deformation modes; (**b**) relative error of different models in predicting uniaxial deformation mode; (**c**) relative error of different models in predicting equibiaxial deformation mode; (**d**) relative error of different models in predicting the deformation mode of pure shear. Note: Only the top three models are presented in Figure 11b–d. $\bar{\delta }$ means the average relative error.

Due to the unique asymmetry and nonlinearity presented by the deformation data from human brain tissue [41], there are currently few constitutive models that can accurately capture it without losing convexity [25]. Applying the modified model proposed in this study to a dataset from human brain tissue will further validate the potential of this modified model in the biomechanical modeling of brain tissue. Table 6 and Figure 11 show some of the results obtained by applying the modified constitutive model proposed in this study to a dataset reflecting the deformation of human brain cortex tissue [41]. From the quantitative indicators in Table 6, it can be found that the total prediction error of the modified constitutive model proposed in this study for the multiaxial deformation of human brain cortex tissue is only 5.6%. Although the error value is slightly higher than the 5.4% of the third-order Ogden model, the difference between the two is only about 3.6%. This difference is smaller when it comes to local indicator—goodness of fit. Specifically, the difference in goodness of fit between the proposed modified model and the third-order Ogden model for uniaxial tension and compression of brain tissue is only 0.1%, while the difference in goodness of fit between simple shear is only 0.4%. Based on these small differences, we believe that our modified model has a comparable ability to describe the multiaxis deformations of human brain cortex tissue to the third-order Ogden model. As shown in Figure 11a,b, our proposed modified model appropriately captures the asymmetry of brain cortex tissue during uniaxial tension and compression, as well as its high nonlinearity during simple shear. Moreover, the relative error of our modified model is also on par with that of the third-order Ogden model (see Figure 11c,d).

In [25], the researcher generalized the Anssari–Benam model, and the first-order form of the obtained model (we call it the generalized Anssari–Benam model) also has a good effect on describing the multiaxial deformation of brain cortex tissue without losing convexity. Because the dataset we used here is the same as that used by the aforementioned researcher (see Table S6 for details), we directly used the parameters of the model obtained by the aforementioned researcher to obtain the quantitative results of the generalized Anassari–Benam model in describing the deformation of brain cortex tissue. It is evident that our proposed modified model outperforms the generalized Anssari–Benam model in describing the multiaxis deformation of brain cortex tissue, both globally and locally (see Table 6). Here, we need to emphasize that when we reproduced the prediction performance of the generalized Anssari–Benam model based on the same parameters and dataset, we found that there is a deviation between its corresponding peak of relative error and that of [25]. The peaks of relative error obtained by us for both uniaxial tension and pure shear slightly exceed 25%. Anyway, our conclusion is consistent with [25], that is, the third-order Ogden model has improved prediction performance compared to the generalized Anssari–Benam model and the performance of our proposed modified model is comparable to the third-order Ogden model; so, our model should still be better than the generalized Anssari–Benam model proposed in [25]. In the next section, we also confirm that our proposed modified model remains stable and convex. In Table 6, we also provide the results of the modified Anssari–Benam model. Figure 11c,d also presents its relative error curves. Based on these results, it is clear that our modified model is still better.

#### 3.1.3. Predictive Ability of the Modified Model to the Untested Deformation Mode

The Treloar’s dataset on natural vulcanized rubber is the most commonly used dataset in verifying the ability of constitutive models to describe untested deformations [15,18,27,30,31,32]. Considering this, we first utilized this dataset to demonstrate the ability of our proposed modified model to predict untested deformations. But, to avoid controversy, here we will mainly use data from UT and PS to calibrate the model, and verify the predictive ability of the obtained model for the untested equibiaxial deformation mode. As introduced in the introduction, this is also a common situation in practice, where the experiments of uniaxial tension and pure shear are easier for researchers compared to the experiments of equibiaxial tension.

**Figure 12:**
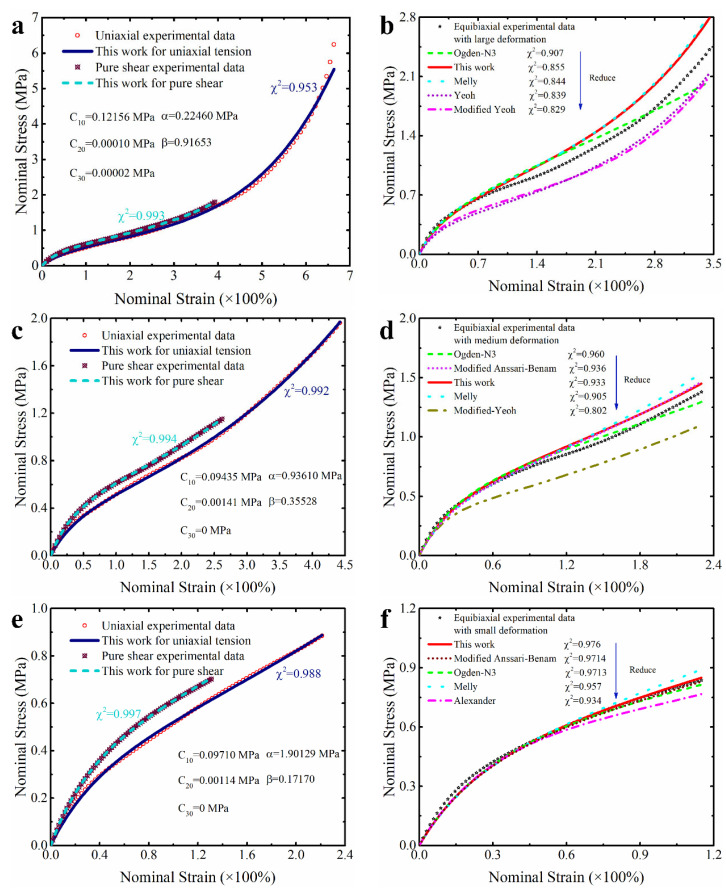
The prediction effects of the modified model for different deformation modes with different deformation ranges: (**a**,**b**) in large deformation; (**c**,**d**) in medium deformation; (**e**,**f**) in small deformation. Here, the parameters of models are calibrated by the experimental data of uniaxial tension and pure shear with different deformation ranges. Only the top five constitutive models are listed on the right side of the figure.

Maintaining consistency with Section 3.1.2 the experimental data of UT and PS from different deformation ranges of this dataset are applied to complete the parameter identification of constitutive models, respectively. The corresponding results are shown in Table 7 and Figure 12 below. Combining these quantitative and qualitative results, it can be seen that the proposed modified constitutive model not only has a good, predicted capability for the modes of deformation which have been tested such as uniaxial tension and pure shear, but can also effectively predict the equibiaxial mode of deformation which is untested. Specifically, the modified model has better predictive ability for the untested equibiaxial deformation mode in different deformation ranges than all models except for the third-order Ogden model in Table 7. In fact, like the other studies mentioned above [15,18,27,30,31,32], if only Treloar’s data from uniaxial tension were used to calibrate these models, the effectively predictive ability of the proposed model for the other two untested deformation modes (ET and PS) would be superior to all models in the comparative list, including the third-order Ogden model (as shown in Table S15 and Figure S1 in the supporting information). This shows that the addition of the correction term not only does not affect the ability of the original Yeoh model to effectively predict untested deformations, but further improves its ability in the modified model.

Table 8 further summarizes the quantitative prediction results of our modified model calibrated by using data of UT and PS from the other four datasets (the corresponding prediction curve is shown in Figure S2). Obviously, the proposed modified constitutive model can also effectively predict the untested equibiaxial deformation of isoprene vulcanized rubber, unfilled silicone rubber, poly-arcylamide hydrogel and carbon-black-filled styrene butadiene rubber. Except for a higher total predictive error for the unfilled silicone rubber, the proposed modified model has a total predictive error of less than 5% for the other three types of rubber. Moreover, compared to other models, these results are also superior to most other models (as shown in Tables S19–S22 in the supporting information), especially the several existing improved models of the Yeoh model. It should be clarified that our modified model is not the only one with a relatively low effective prediction accuracy for untested equibiaxial deformation of the unfilled silicone rubber. As shown in Table S20 (see the supporting information), compared to our proposed modified model, the widely recognized third-order Ogden model and Alexander model with good performance have lower effective prediction accuracy for untested equibiaxial deformation modes of the unfilled silicone rubber. And, the Anssari–Benam model and the modified Anssari–Benam model are directly unable to effectively predict the untested equibiaxial deformation of the unfilled silicone rubber. Although the results show that the modified Yeoh model and the generalized Yeoh model have slightly high prediction accuracy for the untested equibiaxial deformation of the unfilled silicone rubber, their prediction accuracy for UT is not high such that their overall prediction accuracy is not excellent (with a total error of over 8%).

Anyway, in view of the above analysis, it can be believed that it is worthwhile to modify the Yeoh model by the proposed correction term in this study. The correction term expressed in the form of power function based on the principal stretches gives the modified model improved ability in predicting the untested equibiaxial deformation, which means that the proposed modified constitutive model can be applied to effectively predict the equibiaxial mode of deformation when the equibiaxial tension cannot be completed under limited hardware conditions.

### 3.2. The A Posteriori Check of the Modified Model

**Figure 13:**
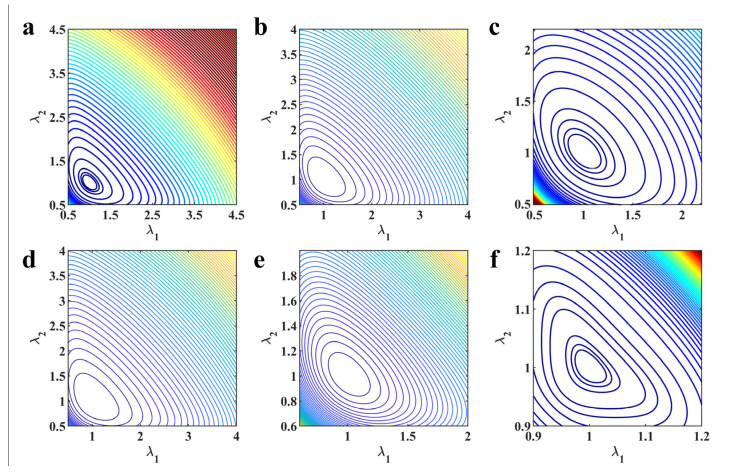
Iso-energy contour plots of the modified models with different calibrated parameters obtained based on different material datasets: (**a**) the dataset of natural vulcanized rubber containing medium deformation; (**b**) the dataset of isoprene vulcanized rubber; (**c**) the dataset of unfilled silicone rubber; (**d**) the dataset of poly-acrylamide hydrogel; (**e**) the dataset of carbon-black-filled styrene butadiene rubber; (**f**) the dataset of human brain cortex tissue.

Possessing the excellent ability to characterize the hyperelastic behavior is only a basic requirement for a constitutive model. If the constitutive model can be further used, it must obey the laws of thermodynamics, and it must also ensure that it has a solution to the boundary-value problem (as explained in Section 2.2). Accordingly, the a posteriori check on the proposed modified model is performed. The a posteriori check is conducted within the range of minimum to maximum principal stretch of the corresponding material datasets ($\lambda_{\min}\leq \lambda \leq \lambda_{\max}$). It is verified that the proposed modified constitutive model calibrated in the above subsection satisfies the inequalities of (12) and can make the matrix of ***D*** positive definite. This means that the modified model with calibrated parameters has a posteriori poly-convexity and Drucker stability. For a more intuitive display, Figure 13 demonstratively presents the iso-energy contour plots of the modified models with different calibrated parameters obtained in Section 3.1.2. It is clear that the stationary points are positioned in the undeformed state ($\lambda_{1}=\lambda_{2}=1$), namely, the modified models with different calibrated parameters obtained based on different material datasets are stable and possess the convexity [4,52].

### 3.3. Validation of the Parameter Identification Method

In order to validate the validity of the parameter identification method introduced in this study, several algorithms that have been used (including the damped least squares algorithm (L-M) [48], genetic algorithm (GA) [50], pattern search algorithm (PS) and the hybrid algorithm (PS-LM) consisting of pattern search and damped least squares [51]) are used to compare with the cyclic genetic-pattern search algorithm (CGA-PS) introduced in this study. These algorithms are used to identify the parameters of the third-order Ogden model and the modified constitutive model, respectively. The dataset used in the identification process is the dataset from the natural vulcanized rubber containing the large deformation. Considering that the L-M algorithm and the PS algorithm are affected by the initial value, the initial values of the L-M algorithm, the PS algorithm, and the PS-LM algorithm will be randomly generated by a random function within five initial intervals of 0–1, 0–0.5, 0–0.25, 0–0.1 and 0–0.05, respectively. And two different initial values are generated in each interval, which means that each algorithm needs to run independently twice with two different initial values within the corresponding interval. Therefore, each algorithm will run independently ten times due to being randomly assigned ten initial values separately. Owing to the inherent random search property, the initial population interval of the GA algorithm and the CGA-PS algorithm is fixed at 0–1, and they will also run ten times independently. Finally, for the ten sets of running results of each algorithm, only the set of results with the minimum total error ($Error_{\mathrm{total}}$, see Equation (17)) will be recorded.

**Figure 14:**
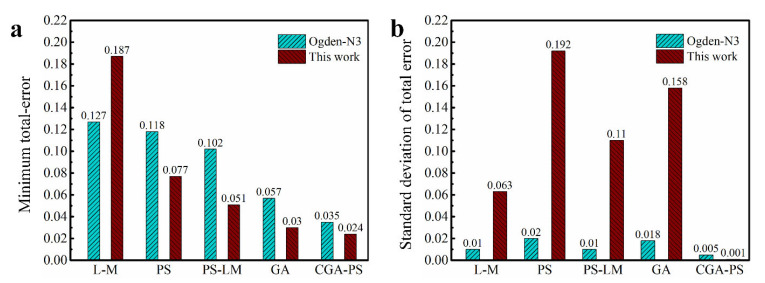
The comparison of different parameter identification methods: (**a**) the minimum total-error after ten runs of different parameter identification methods; (**b**) the standard deviation of total error after ten runs of different parameter identification methods.

**Figure 15:**
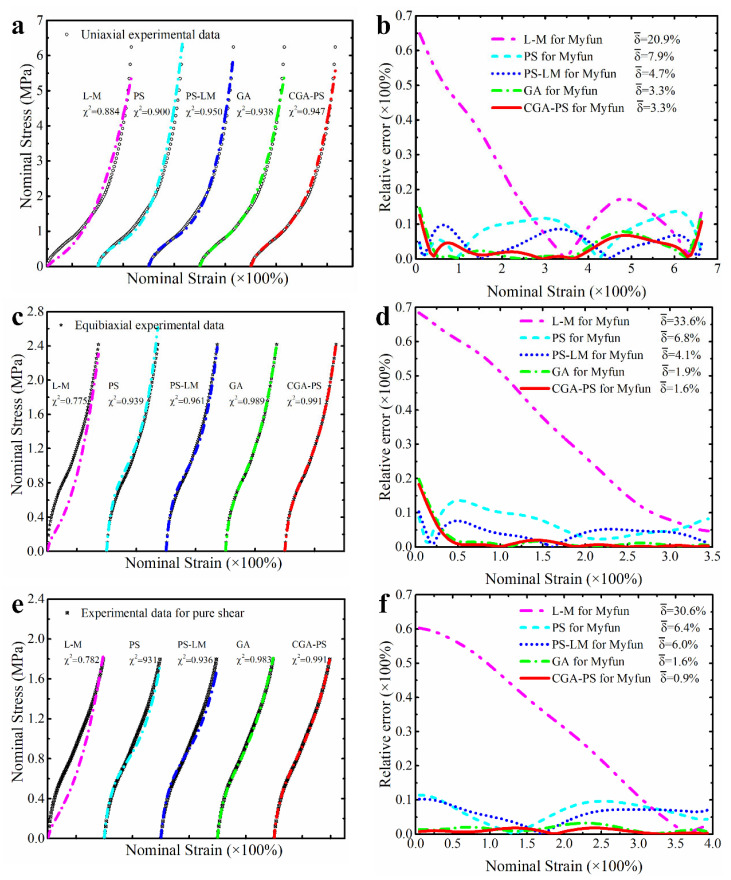
The comparison of prediction performance by the modified model based on different methods of parameter identification: (**a**,**b**) the prediction performance of uniaxial tension by the modified model based on different methods of parameter identification; (**c**,**d**) the prediction performance of equibiaxial tension by the modified model based on different methods of parameter identification; (**e**,**f**) the prediction performance of pure shear by the modified model based on different methods of parameter identification.

According to the minimum total error shown in Figure 14a, it can be seen that the parameter identification method based on the cyclic genetic-pattern search algorithm introduced in this study significantly improves the performance of constitutive models for characterizing hyperelastic properties. In view of the standard deviation of the total error shown in Figure 14b, the parameter identification method introduced in this study has the minimum standard deviation compared with other methods. So, there is reason to believe that the introduced method of parameter identification performs better in terms of uniform convergence. Figure 15 presents the prediction performance of different modes of deformation by the modified model based on different methods of parameter identification (the relevant prediction curve of the third-order Ogden model is shown in Figure S3). It is clear that the introduced method of parameter identification based on the cyclic genetic-pattern search algorithm (CGA-PS) in this study has better performance, which can make the final calibrated models have better characterization capability for different modes of deformation. In addition, we also compared the identification ability of the introduced parameter identification method and the parameter identification method integrated in ABAQUS by the three models of the Arruda–Boyce model, Ogden model and Yeoh model. This comparison procedure uses the experimental data consistent with the above. From this result (as shown in Table 9), it can be found that the introduced identification method also slightly outperforms the parameter identification method integrated in ABAQUS. Hence, one can see that the introduced method of CGA-PS is not only effective, but also has better performance in the process of parameter identification for the hyperelastic constitutive model.

## 4. Conclusions

This paper presents a modified hyperelastic constitutive model based on the first strain invariant and the principal stretches. In order to more accurately identify the parameters of model, a special method of parameter identification based on the cyclic genetic-pattern search algorithm has also been introduced. Combining the experiment data of different rubber materials with the introduced parameter identification method, the performance of the proposed modified constitutive model is fully evaluated. The results show that the proposed modified model not only possesses a significantly improved ability to predict multiaxial deformation, but also has a wider range of material applicability. This advantage is not only reflected in the comparison with the original Yeoh model, but also in the comparison with other improved Yeoh models (such as the modified Yeoh model, the generalized Yeoh model and the Melly model). Even compared with the excellent third-order Ogden model and Alexander model, our proposed modified model with only five undetermined parameters is not necessarily inferior in characterizing the multiaxial deformation of rubber materials. For example, the overall prediction accuracy of the modified model for isoprene vulcanized rubber and poly-acrylamide hydrogel is slightly better than that of the third-order Ogden model and Alexander model. Anyway, for the five rubber materials used in this study, our modified model has a similar level of total prediction accuracy as the two models of the third-order Ogden model and Alexander model (total error is less than 5%). Furthermore, our modified model is also proven to have a good ability to characterize the deformation of human brain cortex tissue. Its prediction accuracy for multiaxial deformations of human brain cortex tissue is not only similar to that of the third-order Ogden model (the third-order Ogden model is considered to be the best model currently characterizing the deformation of the human brain cortex tissue.), but also has no loss of convexity compared to the third-order Ogden model. In addition, the proposed modified model in this study is also proven to hold the improved ability to predict the untested equibiaxial deformation of most rubber materials used in this study. The modified constitutive model calibrated based on different datasets is also verified to have a posteriori Drucker stability and polyconvexity, which lays a foundation for the modified constitutive model to be applied to finite element analysis.

Based on the above conclusions, we believe that the main advantages of the modified constitutive model proposed by us are as follows:

Firstly, although the modified model has five undetermined parameters, it still maintains a relatively simple functional form compared to those models containing logarithmic, exponential or integral expressions, making it easier to perform related mathematical calculations. Having five parameters also ensures that it is neither incompatible with fourth-order weak nonlinear elasticity theory due to having too few parameters, nor does it face difficulties in parameter identification due to having too many parameters.

Secondly, thanks to our proposed correction term based on the principal stretch, this modified constitutive model has significantly improved ability for predicting multiaxial deformation. Moreover, this improved predictive ability has certain universality, that is, the modified model can be applied to various different types of rubber materials (including natural unfilled or filled rubber, silicone rubber and hydrogel). There are currently few studies that apply their model to so many types of material.

Thirdly, this modified constitutive model can, with relative accuracy, predict the multiaxial deformation of human brain cortex tissue (including uniaxial tension, uniaxial compression and simple shear) while ensuring convexity. This gives it the potentiality to characterize the biomechanics of soft biological tissues.

Finally, the modified constitutive model also has an improved capacity to predict untested equibiaxial deformation. This advantage is very useful in the situation where equibiaxial tension cannot be completed under limited hardware conditions.

Compared with other parameter identification methods, the introduced method of parameter identification has been proved not only to take into account different modes of deformation, but also makes models have better performance. The excellent capability of the introduced method of parameter identification benefits from the strong ability of global search of the genetic algorithm and the strong ability of local search of the pattern search algorithm, and the addition of the cyclic structure further weakens its dependence on the initial value, thereby making its uniform convergence better.

## Figures and Tables

**Table 1:** Total error and goodness of fit of different models on natural vulcanized rubber [37].

Models	Large Deformation				Medium Deformation				Small Deformation			
	Error	UT	ET	PS	Error	UT	ET	PS	Error	UT	ET	PS
Ogden-N3	0.035	0.927	0.987	0.981	0.011	0.988	0.986	0.994	0.012	0.988	0.979	0.996
Alexander	0.017	0.982	0.984	0.982	0.016	0.981	0.987	0.985	0.013	0.987	0.980	0.996
Yeoh	0.083	0.928	0.853	0.969	0.093	0.940	0.809	0.973	0.117	0.872	0.810	0.967
Melly	0.033	0.944	0.969	0.988	0.021	0.984	0.967	0.987	0.020	0.986	0.966	0.988
Modified Yeoh	0.077	0.938	0.843	0.989	0.084	0.942	0.809	0.996	0.097	0.929	0.782	0.999
Generalized Yeoh	0.084	0.914	0.861	0.973	0.086	0.943	0.807	0.993	0.110	0.914	0.791	0.967
Anssari-Benam	0.079	0.966	0.828	0.968	0.093	0.941	0.806	0.973	0.099	0.930	0.781	0.993
Modified Anssari–Benam	0.164	0.608	0.954	0.945	0.014	0.986	0.981	0.990	0.013	0.987	0.976	0.997
**This work**	**0.024**	**0.947**	**0.991**	**0.991**	**0.012**	**0.991**	**0.982**	**0.991**	**0.013**	**0.987**	**0.978**	**0.997**

Notes: Error indicates the total error of different models; UT, ET and PS represent the goodness of fit of different models for uniaxial tension, equibiaxial tension and pure shear, respectively. The model parameters are shown in Tables S7–S9 in supporting information.

**Table 2:** Total error and goodness of fit of different models on isoprene vulcanized rubber [38].

Models	The Number of Coefficient	Error	UT	ET	PS
Ogden-N3	6	0.017	0.970	0.996	0.984
Alexander	5	0.025	0.986	0.986	0.954
Yeoh	3	0.092	0.964	0.815	0.944
Melly	4	0.032	0.979	0.976	0.950
Modified Yeoh	5	0.068	0.984	0.845	0.966
Generalized Yeoh	6	0.068	0.987	0.847	0.963
Anssari–Benam	3	0.081	0.987	0.800	0.976
Modified Anssari–Benam	5	0.019	0.980	0.997	0.965
**This work**	**5**	**0.015**	**0.981**	**0.997**	**0.977**

Notes: Error indicates the total error of different models; UT, ET and PS represent the goodness of fit of different models for uniaxial tension, equibiaxial tension and pure shear, respectively. The model parameters are shown in Table S10 in supporting information.

**Table 3:** Total error and goodness of fit of different models on unfilled silicone rubber [17].

Models	The Number of Coefficient	Error	UT	ET	PS
Ogden-N3	6	0.029	0.970	0.985	0.958
Alexander	5	0.044	0.936	0.988	0.943
Yeoh	3	0.233	0.606	0.897	0.800
Melly	4	0.048	0.939	0.963	0.955
Modified Yeoh	5	0.047	0.932	0.961	0.965
Generalized Yeoh	6	0.048	0.931	0.955	0.970
Anssari–Benam	3	0.046	0.922	0.983	0.956
Modified Anssari–Benam	5	0.044	0.934	0.986	0.947
**This work**	**5**	**0.044**	**0.920**	**0.972**	**0.978**

Notes: Error indicates the total error of different models; UT, ET and PS represent the goodness of fit of different models for uniaxial tension, equibiaxial tension and pure shear, respectively. The model parameters are shown in Table S11 in supporting information.

**Table 4:** Total error and goodness of fit of different models on poly-arcylamide hydrogel [39].

Models	The Number of Coefficient	Error	UT	ET	PS
Ogden-N3	6	0.013	0.982	0.992	0.988
Alexander	5	0.014	0.981	0.993	0.986
Yeoh	3	0.090	0.966	0.778	0.986
Melly	4	0.012	0.981	0.995	0.987
Modified Yeoh	5	0.089	0.967	0.777	0.991
Generalized Yeoh	6	0.089	0.965	0.780	0.988
Anssari–Benam	3	0.095	0.956	0.783	0.977
Modified Anssari–Benam	5	0.018	0.984	0.980	0.984
**This work**	**5**	**0.011**	**0.989**	**0.994**	**0.985**

Notes: Error indicates the total error of different models; UT, ET and PS represent the goodness of fit of different models for uniaxial tension, equibiaxial tension and pure shear, respectively. The model parameters are shown in Table S12 in supporting information.

**Table 5:** Total error and goodness of fit of different models on carbon-black-filled styrene butadiene rubber [40].

Models	The Number of Coefficient	Error	UT	ET	PS
Ogden-N3	6	0.037	0.968	0.966	0.954
Alexander	5	0.023	0.975	0.975	0.982
Yeoh	3	0.106	0.887	0.885	0.909
Melly	4	0.049	0.958	0.960	0.936
Modified Yeoh	5	0.080	0.916	0.887	0.956
Generalized Yeoh	6	0.079	0.914	0.883	0.965
Anssari–Benam	3	0.107	0.890	0.875	0.914
Modified Anssari–Benam	5	0.039	0.963	0.965	0.954
**This work**	**5**	**0.034**	**0.967**	**0.969**	**0.961**

Notes: Error indicates the total error of different models; UT, ET and PS represent the goodness of fit of different models for uniaxial tension, equibiaxial tension and pure shear, respectively. The model parameters are shown in Table S13 in supporting information.

**Table 6:** Total error and goodness of fit of different models on human brain cortex tissue [41].

Models	The Number of Coefficient	Error	UT	SS
Ogden-N3	6	0.054	0.927	0.966
Generalized Anssari–Benam	4	0.083	0.922	0.913
Modified Anssari–Benam	5	0.060	0.915	0.965
**This work**	**5**	**0.056**	**0.926**	**0.962**

Notes: Error indicates the total error of different models; UT, ET and PS represent the goodness of fit of different models for uniaxial tension, equibiaxial tension and pure shear, respectively. The model parameters are shown in Table S14 in supporting information.

**Table 7:** The predictive results of different models calibrated by the data from UT and PS of natural vulcanized rubber [37].

Model	Large Deformation				Medium Deformation				Small Deformation			
	Error	UT	ET	PS	Error	UT	ET	PS	Error	UT	ET	PS
Ogden-N3	0.043	0.975	0.907	0.989	0.018	0.991	0.960	0.995	0.014	0.988	0.971	0.997
Alexander	0.175	0.983	0.501	0.991	0.111	0.983	0.689	0.993	0.027	0.988	0.934	0.998
Yeoh	0.086	0.933	0.839	0.971	0.095	0.952	0.794	0.968	0.128	0.941	0.756	0.918
Melly	0.069	0.957	0.844	0.993	0.039	0.988	0.905	0.989	0.022	0.987	0.957	0.989
Modified Yeoh	0.079	0.949	0.829	0.986	0.086	0.948	0.802	0.993	0.098	0.936	0.774	0.995
Generalized Yeoh	0.086	0.970	0.800	0.973	0.092	0.954	0.787	0.983	0.108	0.961	0.757	0.959
Anssari–Benam	0.082	0.972	0.813	0.971	0.096	0.952	0.790	0.970	0.100	0.932	0.773	0.995
Modified Anssari–Benam	0.225	0.626	0.757	0.942	0.029	0.988	0.936	0.991	0.015	0.986	0.971	0.998
**This work**	**0.066**	**0.953**	**0.855**	**0.993**	**0.027**	**0.992**	**0.933**	**0.994**	**0.013**	**0.988**	**0.976**	**0.997**

Notes: “Error” indicates the total error in predicting the three modes of deformation. UT, ET and PS represent the goodness of fit of different models for uniaxial tension, equibiaxial tension and pure shear, respectively. The model parameters are shown in Tables S16–S18 in supporting information.

**Table 8:** The predictive results of the modified model calibrated by the data from UT and PS of others rubber.

Different Materials	Error	UT	ET	PS
Isoprene vulcanized rubber	0.041	0.974	0.911	0.993
Unfilled silicone rubber	0.134	0.982	0.642	0.974
Poly-arcylamide hydrogel	0.035	0.992	0.914	0.990
Carbon-black-filled styrene butadiene rubber	0.049	0.968	0.938	0.949

Notes: “Error” indicates the total error in predicting the three modes of deformation. UT, ET and PS represent the goodness of fit of different models for uniaxial tension, equibiaxial tension and pure shear, respectively. The model parameters are shown in Tables S23–S26 in supporting information.

**Table 9:** The comparison between the ability of the introduced parameter identification method and the ability of parameter identification method integrated in ABAQUS.

Method	Arruda–Boyce Model	Third-Order Ogden Model	Yeoh Model
ABAQUS	*Error* = 0.103	*Error* = 0.041	*Error* = 0.083
	$\chi_{UT}^{2}$ = 0.894	$\chi_{UT}^{2}$ = 0.925	$\chi_{UT}^{2}$ = 0.932
	$\chi_{ET}^{2}$ = 0.877	$\chi_{ET}^{2}$ = 0.978	$\chi_{ET}^{2}$ = 0.858
	$\chi_{PS}^{2}$ =0.920	$\chi_{PS}^{2}$ = 0.974	$\chi_{PS}^{2}$ = 0.962
This work	*Error* = 0.087	*Error* = 0.035	*Error* = 0.077
	$\chi_{UT}^{2}$ = 0.934	$\chi_{UT}^{2}$ = 0.927	$\chi_{UT}^{2}$ = 0.943
	$\chi_{ET}^{2}$ = 0.852	$\chi_{ET}^{2}$ = 0.987	$\chi_{ET}^{2}$ = 0.839
	$\chi_{PS}^{2}$ = 0.955	$\chi_{PS}^{2}$ = 0.981	$\chi_{PS}^{2}$ = 0.988

Note: The *Error* is the total prediction error, $\chi_{UT}^{2}$ is the goodness of fit for the uniaxial tension, $\chi_{ET}^{2}$ is the goodness of fit for the equibiaxial tension, and $\chi_{PS}^{2}$ is the goodness of fit for the pure shear. The model parameters are shown in Table S27 in supporting information.

## Data Availability

All data from this study are presented in the paper.

## Supplementary Materials

The following supporting information can be downloaded at https://www.mdpi.com/article/10.3390/polym15153172/s1.
