# Supplementary material for: A Modified Constitutive Model for Isotropic Hyperelastic Polymeric Materials and Its Parameter Identification
Source: Polymers (Basel). 2023 Jul 26;15(15):3172. doi: 10.3390/polym15153172 (PMC10421088; doi:10.3390/polym15153172)
Supplement: Supplementary file 1 [file polymers-15-03172-s001.zip › Supporting Information.pdf]

Supporting Information

A modified constitutive model for isotropic hyperelastic polymeric materials and its parameter identification

Wei Wang • Yang Liu* • Zongwu Xie

Table S1 The experimental data for natural unfilled vulcanized rubber from Treloar [24].

Uniaxial tension		Equal-biaxial tension		Pure shear	
λ	T/MPa	λ	T/MPa	λ	T/MPa
1.0000	0.0000	1.0000	0.0000	1.0000	0.0000
1.1151	0.1135	1.0270	0.0926	1.0865	0.0992
1.2302	0.1987	1.0650	0.1566	1.1514	0.1839
1.3646	0.2839	1.1150	0.2371	1.2739	0.2744
1.5951	0.3785	1.1400	0.2570	1.4036	0.3649
1.8641	0.4731	1.2000	0.3258	1.8072	0.5431
2.1523	0.5583	1.3100	0.4339	2.3622	0.7214
2.4020	0.6434	1.4200	0.5079	2.9460	0.9056
2.9977	0.8232	1.6800	0.6470	3.4432	1.0926
3.5549	1.0124	1.9400	0.7624	3.9333	1.2621
3.9967	1.1922	2.4900	0.9590	4.3369	1.4462
4.7456	1.5612	3.0300	1.2382	4.6685	1.6185
5.3600	1.9113	3.4300	1.4387	4.9279	1.7966
5.7629	2.2709	3.7500	1.7052		
6.1657	2.6494	4.0700	1.9704		
6.4147	2.9995	4.2600	2.2001		
6.6252	3.3685	4.4500	2.4160		
6.8742	3.7281				
7.0654	4.0971				
7.1798	4.4472				
7.2942	4.8257				
7.4470	5.1947				
7.5229	5.5542				
7.6362	6.2828				

Note: The specific data is from the literature (Li, X.B. and Wei, Y.T.: Classic strain energy functions and constitutive tests of rubber-like materials. Rubber Chemistry & Technology 88(4), 604-627 (2015)). The stress T in the table is nominal stress. The equidistant data points with different deformation ranges from the dataset are shown in the file--Treloar.xlsx.

Table S2 The experimental data for isoprene vulcanized rubber from Kawabata[49]

Uniaxial tension		Equal-biaxial tension		Pure shear	
λ	σ/MPa	λ	σ/MPa	λ	σ/MPa
1.00	0	1.00	0	1.00	0
1.04	0.045	1.04	0.088	1.04	0.061
1.06	0.065	1.06	0.129	1.06	0.091
1.08	0.093	1.08	0.178	1.08	0.122
1.10	0.117	1.10	0.218	1.10	0.152
1.12	0.140	1.12	0.257	1.12	0.181
1.14	0.163	1.14	0.293	1.14	0.209
1.16	0.186	1.16	0.329	1.16	0.238
1.20	0.235	1.20	0.396	1.20	0.294
1.24	0.282	1.24	0.461	1.24	0.349
1.30	0.351	1.30	0.547	1.30	0.434
1.60	0.693	1.60	0.976	1.60	0.819
1.90	1.051	1.90	1.414	1.90	1.193
2.20	1.432	2.20	1.896	2.20	1.593
2.50	1.850	2.50	2.435	2.50	2.022
2.80	2.316	2.80	3.030	2.80	2.482
3.10	2.849	3.10	3.689	3.10	2.973
3.40	3.454			3.40	3.505
3.70	4.133			3.70	4.077

Note: The specific data is from the literature (Afshin Anssari-Benam, Andrea Bucchi. A generalised neo-Hookean strain energy function for application to the finite deformation of elastomers[J]. International Journal of Non-Linear Mechanics, 2021, 128:103626.). The stress σ in the table is true stress.

Table S3 The experimental data for unfilled silicone rubber from Meunier[17]

Uniaxial tension		Equal-biaxial tension		Pure shear	
λ	σ/MPa	λ	σ/MPa	λ	σ/MPa
0.49	-0.68	1	0	1	0
0.52	-0.62	1.05	0.10	1.02	0.05
0.55	-0.56	1.10	0.20	1.05	0.10
0.58	-0.51	1.18	0.32	1.10	0.17
0.61	-0.46	1.23	0.41	1.15	0.23
0.64	-0.41	1.34	0.54	1.22	0.30
0.67	-0.37	1.44	0.69	1.29	0.39
0.71	-0.33	1.54	0.83	1.38	0.48
0.74	-0.29	1.68	1.09	1.48	0.59
0.77	-0.26	1.77	1.31	1.57	0.71
0.80	-0.22	1.80	1.39	1.66	0.83
0.83	-0.18	1.84	1.52	1.75	0.96
0.86	-0.15	1.95	1.97	1.83	1.10
0.89	-0.11	2.06	2.72	1.90	1.24
0.93	-0.08			1.97	1.38
0.96	-0.04			2.02	1.51
1.00	0			2.07	1.66
1.05	0.05			2.12	1.80
1.11	0.11			2.15	1.94
1.18	0.17				
1.27	0.25				
1.36	0.33				
1.46	0.44				
1.56	0.54				
1.66	0.66				
1.76	0.78				
1.85	0.91				
1.93	1.06				
1.99	1.18				
2.04	1.32				
2.09	1.45				
2.14	1.60				
2.17	1.73				

Note: The specific data is from the literature (Afshin Anssari-Benam, Andrea Bucchi. A generalised neo-Hookean strain energy function for application to the finite deformation of elastomers[J]. International Journal of Non-Linear Mechanics, 2021, 128:103626.). The stress σ in the table is true stress.

Table S4 The experimental data for poly-acrylamide hydrogel from Yohsuke[50]

Uniaxial tension		Equal-biaxial tension		Pure shear	
λ	σ/KPa	λ	σ/KPa	λ	σ/KPa
1.00	0	1.00	0	1.00	0
1.06	1.08	1.06	2.06	1.06	1.67
1.13	2.20	1.13	4.08	1.13	2.93
1.25	4.17	1.25	7.19	1.25	5.10
1.38	6.28	1.38	10.51	1.38	7.69
1.54	9.16	1.54	14.60	1.54	10.73
1.72	12.24	1.72	18.92	1.72	14.47
1.91	16.08	1.91	24.57	1.91	18.56
2.10	20.04	2.10	30.74	2.10	22.76
2.29	24.63	2.29	37.37	2.29	27.39
2.47	29.10	2.47	44.68	2.47	32.54
2.72	36.23			2.72	39.25
3.03	46.581				
3.32	57.23				
3.60	69.59				
4.00	91.85				

Note: The specific data is from the literature (Afshin Anssari-Benam. Large isotropic elastic deformations: on a comprehensive model to correlate the theory and experiments for incompressible rubber-like materials[J]. Journal of Elasticity, 2023, 153:219-244.). The stress σ in the table is Cauchy stress.

Table S5 The experimental data for carbon-black-filled styrene butadiene rubber from Fujikawa[51]

Uniaxial tension		Equal-biaxial tension		Pure shear	
λ	σ/MPa	λ	σ/MPa	λ	σ/MPa
1	0	1	0	1	0
1.05	0.32	1.04	0.52	1.04	0.42
1.11	0.59	1.09	0.94	1.09	0.74
1.16	0.81	1.14	1.26	1.14	1.00

1.21	0.99	1.18	1.53	1.19	1.21
1.26	1.18	1.23	1.75	1.23	1.41
1.31	1.33	1.28	2.00	1.28	1.60
1.36	1.48	1.32	2.25	1.33	1.78
1.41	1.65	1.37	2.49	1.38	2.00
1.46	1.85	1.42	2.79	1.42	2.17
1.51	2.00	1.46	3.11	1.47	2.37
1.56	2.22	1.51	3.43	1.52	2.59
1.60	2.42	1.56	3.75	1.57	2.81
1.65	2.62	1.61	4.05	1.62	3.04
1.72	2.81	1.66	4.34	1.66	3.23
1.74	2.99	1.70	4.54	1.71	3.41
1.79	3.11			1.76	3.55

Note: The specific data is from the literature (Afshin Anssari-Benam. Comparative modelling results between a separable and a non-separable form of principal stretches-based strain energy functions for a variety of isotropic incompressible soft solids: Ogden mdel compared with a parent model[J]. Mechanics of Soft Materials, 2023, 5:2.). The stress σ in the table is true stress.

Table S6 The experimental data for human brain tissue from Budday[52]

Uniaxial tension		Simple shear	
λ	σ/KPa	γ	σ/KPa
0.90	-1.06	-0.20	-0.55
0.91	-0.82	-0.175	-0.38
0.92	-0.62	-0.15	-0.27
0.94	-0.46	-0.125	-0.19
0.95	-0.33	-0.10	-0.14
0.96	-0.225	-0.075	-0.1
0.975	-0.135	-0.05	-0.06
0.99	-0.06	-0.02	-0.04
1.00	0	0	0
1.01	0.04	0.02	0.03
1.025	0.08	0.05	0.06
1.04	0.12	0.07	0.1
1.05	0.15	0.10	0.14
1.06	0.19	0.12	0.19
1.075	0.25	0.15	0.27
1.09	0.34	0.17	0.37
1.10	0.46	0.20	0.54

Note: The specific data is from the literature (Afshin Anssari-Benam, Michel Destrade, Giuseppe Saccomandi. Modelling brain tissue elasticity with the Ogden model and an alternative family of constitutive models[J]. Philosophical Transactions A, 2022, 380:20210325). The stress σ in the table is true stress.

Table S7. The model parameters for large deformation in Table 1

Model	Parameters
Ogden-N3	$\alpha_1=4.720;\ \mu_1=0.005$ MPa; $\alpha_2=1.334;\ \mu_2=0.357$ MPa; $\alpha_3=-1.812;\ \mu_3=0.016$ MPa;
Alexander	$C_1=0.139$ MPa; $C_2=0.320$ MPa; $C_3=0.002$ MPa; $k=0.000338;\ \gamma=12.10744;$
Yeoh	$C_{10}=0.152$ MPa; $C_{20}=0$ MPa; $C_{30}=0.0000228$ MPa;
Melly	$C_{10}=0.137$ MPa; $C_{20}=0$ MPa; $C_{30}=0.0000235$ MPa; $D=0.094$ MPa;
Modified Yeoh	$C_{10}=1.44E-7$ MPa; $C_{20}=0.00148$ MPa; $C_{30}=0.0000189$ MPa; $\alpha=0.165$ MPa; $\beta=0.0288;$
Generalized Yeoh	$K_1=1.2278$ MPa; $m=1;\ K_2=-1.0309$ MPa; $p=1.01847;\ K_3=0.000392$ MPa; $q=2.3972;$
Anssari-Benam	$\mu=0.29086$ MPa; $N=24.9907;\ n=2.0108;$
Modified Anssari-Benam	$\mu=1.1692$ MPa; $N=0.05779;\ n=3.35977;\ \alpha=0.003166$ MPa; $\beta=2.3647;$
My work	$C_{10}=0.112895$ MPa; $C_{20}=0.00041$ MPa; $C_{30}=0.000021669$ MPa; $\alpha=0.792239$ MPa; $\beta=0.3361846;$

Table S8. The model parameters for medium deformation in Table 1

Model	Parameters
Ogden-N3	$\alpha_1=0.39472;\ \mu_1=0.268358$ MPa; $\alpha_2=3.010134;\ \mu_2=0.081632$ MPa; $\alpha_3=-1.88910;\ \mu_3=0.00923$ MPa;
Alexander	$C_1=0.132$ MPa; $C_2=0.262585$ MPa; $C_3=0.003018$ MPa; $k=0.00045775;\ \gamma=6.62166;$
Yeoh	$C_{10}=0.1575635$ MPa; $C_{20}=0$ MPa; $C_{30}=0.000010285$ MPa;
Melly	$C_{10}=0.13631957$ MPa; $C_{20}=0$ MPa; $C_{30}=0.0000203$ MPa; $D=0.1120827$ MPa;
Modified Yeoh	$C_{10}=0.148625$ MPa; $C_{20}=0.0004428$ MPa; $C_{30}=0.0000039786$ MPa; $\alpha=0.032364$ MPa; $\beta=0.47151486;$
Generalized Yeoh	$K_1=0.1917369$ MPa; $m=1;\ K_2=-0.072778$ MPa; $p=1.5876;\ K_3=0.0554836$ MPa; $q=1.65405;$

Anssari-Benam	μ=0.313768 MPa; N=12.41387; n=1.04495;
Modified Anssari-Benam	μ=0.215047 MPa; N=25.51408; n=85.0; α=0.6880299 MPa; β=0.37208;
My work	C_{10}=0.0938605 MPa; C_{20}=0.00138968 MPa; C_{30}=0 MPa; α=3.6362 MPa; β=0.09307;

Table S9. The model parameters for small deformation in Table 1

Model	Parameters
Ogden-N3	α_1=-0.42082; μ_1=0.094627 MPa; α_2=2.822296; μ_2=0.096908 MPa; α_3=0.56605; μ_3=0.1682697 MPa;
Alexander	C_1=0.125446 MPa; C_2=0.180328 MPa; C_3=0.007073 MPa; k=0.000964878; γ=3.7760124;
Yeoh	C_{10}=0.16289 MPa; C_{20}=0 MPa; C_{30}=0 MPa;
Melly	C_{10}=0.131834 MPa; C_{20}=0 MPa; C_{30}=0 MPa; D=0.14487 MPa;
Modified Yeoh	C_{10}=0.009 MPa; C_{20}=0.002555 MPa; C_{30}=0.0000001147 MPa; α=0.16779856 MPa; β=0.07253678;
Generalized Yeoh	K_1=0.46885 MPa; m=1; K_2=-0.4695 MPa; p=1.122683; K_3=0.186023 MPa; q=1.22587;
Anssari-Benam	μ=0.37157 MPa; N=42.787; n=0.26312;
Modified Anssari-Benam	μ=0.19653 MPa; N=13.6234; n=3.10978; α=1.236187 MPa; β=0.2478425;
My work	C_{10}=0.09095 MPa; C_{20}=0.00133 MPa; C_{30}=0 MPa; α=8.7237 MPa; β=0.04076;

Table S10. The model parameters for isoprene vulcanized rubber in Table 2

Model	Parameters
Ogden-N3	α_1=-1.5722; μ_1=0.01719 MPa; α_2=10.5078; μ_2=0.000001 MPa; α_3=1.6233; μ_3=0.37751 MPa;
Alexander	C_1=0.1444 MPa; C_2=0.16637 MPa; C_3=0.00337 MPa; k=0; γ=3.535169;
Yeoh	C_{10}=0.1575 MPa; C_{20}=0 MPa; C_{30}=0.00000259 MPa;
Melly	C_{10}=0.147067 MPa; C_{20}=0 MPa; C_{30}=0 MPa; D=0.095079 MPa;
Modified Yeoh	C_{10}=0.0056 MPa; C_{20}=0.000337 MPa; C_{30}=0.00011 MPa; α=0.1860795 MPa; β=0.05467;
Generalized Yeoh	K_1=1.16528 MPa; m=1; K_2=-0.97185 MPa; p=1.01405; K_3=0.0000001 MPa; q=5.08984;
Anssari-Benam	μ=0.22303 MPa; N=0.601714; n=0.751607;
Modified Anssari-Benam	μ=0.24728 MPa; N=0.6888547; n=0.897568; α=0.191818 MPa; β=0.76695;
My work	C_{10}=2.494 MPa; C_{20}=0.0034 MPa; C_{30}=-0.00004348 MPa; α=4.475518 MPa; β=-2.053077;

Table S11. The model parameters for unfilled silicone rubber in Table 3

Model	Parameters
Ogden-N3	α_1=0.3587; μ_1=0.28225 MPa; α_2=-6.45; μ_2=0.000245 MPa; α_3=5.6138; μ_3=0.036298 MPa;
Alexander	C_1=0.16297 MPa; C_2=0.0261936 MPa; C_3=0 MPa; k=0.021613; γ=1.672377859;
Yeoh	C_{10}=0.10936 MPa; C_{20}=0.047917 MPa; C_{30}=0 MPa;
Melly	C_{10}=0.16308897 MPa; C_{20}=0 MPa; C_{30}=0.00159675 MPa; D=0.0141 MPa;
Modified Yeoh	C_{10}=0.155636 MPa; C_{20}=0.00338289 MPa; C_{30}=0.0013389 MPa; α=0.03732 MPa; β=2.509305;
Generalized Yeoh	K_1=0.90577 MPa; m=1; K_2=-0.737696 MPa; p=1.021726; K_3=0.006919 MPa; q=2.421487;
Anssari-Benam	μ=0.287468 MPa; N=3.716448; n=1.62188;
Modified Anssari-Benam	μ=0.272177 MPa; N=3.658636; n=1.58244; α=0.54795 MPa; β=0.084;
My work	C_{10}=0.17927 MPa; C_{20}=-0.00731 MPa; C_{30}=0.00322 MPa; α=-0.00068986 MPa; β=4.70799;

Table S12. The model parameters for poly-arcylamide hydrogel in Table 4

Model	Parameters
Ogden-N3	α_1=1.99617; μ_1=0.004565 MPa; α_2=-1.26655; μ_2=0.0010889 MPa; α_3=7.226208; μ_3=0.000002788 MPa;
Alexander	C_1=0.0023 MPa; C_2=0.004895 MPa; C_3=0.00008 MPa; k=0.001156; γ=12.0408;
Yeoh	C_{10}=0.0027 MPa; C_{20}=0 MPa; C_{30}=3.874E-7 MPa;
Melly	C_{10}=0.0022 MPa; C_{20}=0.000006824 MPa; C_{30}=8.49E-7 MPa; D=0.0022786 MPa;
Modified Yeoh	C_{10}=0.00251 MPa; C_{20}=0.0000152 MPa; C_{30}=0 MPa; α=0.000321 MPa; β=0.35653;

Generalized Yeoh	K_1=0.050987 MPa; m=1.0755; K_2=-0.0491 MPa; p=1.08738; K_3=0.001006 MPa; q=1.338512;
Anssari-Benam	μ=0.096 MPa; N=0.00618; n=16.68516;
Modified Anssari-Benam	μ=0.004677 MPa; N=7.81967; n=1.09826; α=0.0012 MPa; β=1.369;
My work	C_{10}=0.00187 MPa; C_{20}=0.0000316 MPa; C_{30}=0 MPa; α=0.00628 MPa; β=0.59589;

Table S13. The model parameters for carbon-black-filled styrene butadiene rubber in Table 5

Model	Parameters
Ogden-N3	α_1=0.909289; μ_1=1.25589 MPa; α_2=-4.18085; μ_2=0.0101 MPa; α_3=0.8449; μ_3=0.25837 MPa;
Alexander	C_1=0.49284 MPa; C_2=0.07262 MPa; C_3=0.11140 MPa; k=1.49E-8; γ=0.165188;
Yeoh	C_{10}=0.655183 MPa; C_{20}=0 MPa; C_{30}=0.00693 MPa;
Melly	C_{10}=0.5132 MPa; C_{20}=0 MPa; C_{30}=0 MPa; D=0.67044 MPa;
Modified Yeoh	C_{10}=0.4509 MPa; C_{20}=0.07017 MPa; C_{30}=0 MPa; α=0.49347 MPa; β=2.17857;
Generalized Yeoh	K_1=3.16754 MPa; m=1; K_2=-2.4819 MPa; p=1.05753; K_3=0.046104 MPa; q=2.39379;
Anssari-Benam	μ=1.27325 MPa; N=2.20960; n=1.04265;
Modified Anssari-Benam	μ=1.52426 MPa; N=7.80347; n=0.38067; α=0.379 MPa; β=1.8624;
My work	C_{10}=0.164 MPa; C_{20}=-0.0333 MPa; C_{30}=0.0089348 MPa; α=-4.16605 MPa; β=-0.6066;

Table S14. The model parameters for human brain cortex tissue in Table 6

Model	Parameters
Ogden-N3	α_1=61.2136; μ_1=0.01101 KPa; α_2=-21.351585; μ_2=1.2009 KPa; α_3=2.49218; μ_3=0.00122 KPa;
Generalized Anssari-Benam	μ=0.02 KPa; N=7.52; n=19.99; α=-15.93;
Modified Anssari-Benam	μ=0.01145 KPa; N=1.01874; n=1.46566; α=0.09726 KPa; β=21.49197;
My work	C_{10}=10.3115 KPa; C_{20}=6.07355 KPa; C_{30}=87.4228 KPa; α=9.83259 KPa; β=-3.93031;

Table S15. The predictive results of different models calibrated by the data from UT of natural vulcanized rubber [24].

Model	Large deformation				Medium deformation				Small deformation			
	Error	UT	ET	PS	Error	UT	ET	PS	Error	UT	ET	PS
Ogden-N3	--	0.982	--	--	0.032	0.993	0.928	0.985	--	0.993	--	--
Alexander												
Yeoh	0.131	0.953	0.775	0.880	0.119	0.982	0.751	0.909	0.145	0.965	0.721	0.878
Melly	0.241	0.959	0.421	0.895	0.188	0.993	0.542	0.900	0.082	0.990	0.820	0.946
Biderman	--	0.960	--	0.553	--	0.993	--	0.663	--	0.990	--	0.831
Polynomial-N2	--	0.916	--	--	--	0.993	--	0.568	--	0.992	--	0.208
GeneralizedYeoh	0.095	0.972	0.783	0.960	0.117	0.991	0.751	0.909	0.124	0.994	0.727	0.908
Arruda-Boyce	0.126	0.966	0.773	0.884	0.124	0.977	0.751	0.899	0.145	0.965	0.721	0.878
Exp-Ln	0.127	0.957	0.773	0.889	0.114	0.989	0.751	0.916	0.125	0.986	0.727	0.913
Modified-Yeoh	0.112	0.965	0.779	0.920	0.115	0.993	0.752	0.912	0.128	0.990	0.726	0901
**This work**	**0.041**	**0.960**	**0.966**	**0.952**	**0.022**	**0.989**	**0.959**	**0.987**	**0.026**	**0.988**	**0.949**	**0.987**

Notes: Error indicates the total error in predicting the three modes of deformation; UT, ET and PS represent the goodness of fit of different models for uniaxial tension, equi-biaxial tension and pure shear; “--” indicates that the goodness of fit is negative and the prediction is invalid.

Table S16. The model parameters for large deformation in Table 7

Model	Parameters
Ogden-N3	α_1=2.38743; μ_1=0.17449 MPa; α_2=-0.69033; μ_2=0.16442 MPa; α_3=7.931477; μ_3=0.00001 MPa;
Alexander	C_1=0.14023 MPa; C_2=0.023797 MPa; C_3=0.0118826 MPa; k=0.0003355; γ=0.651445;
Yeoh	C_{10}=0.14971 MPa; C_{20}=0 MPa; C_{30}=0.0000225 MPa;
Melly	C_{10}=0.12486 MPa; C_{20}=3.979E-8 MPa; C_{30}=0.000026179 MPa; D=0.17694 MPa;
Modified Yeoh	C_{10}=2.099E-7 MPa; C_{20}=0.0001419 MPa; C_{30}=0.00002889 MPa; α=0.1643997 MPa; β=0.01107;
Generalized Yeoh	K_1=0.1606 MPa; m=1; K_2=-1.78E-7 MPa; p=1.00064; K_3=0.0000001 MPa; q=4.28993;
Anssari-Benam	μ=0.288323 MPa; N=24.36469; n=1.848835;

Modified Anssari-Benam	*μ=6.033266 MPa; N=0.02078; n=16.496647; α=0.00002889 MPa; β=0.0000419;*
My work	*C_{10}=0.121556 MPa; C_{20}=0.0001045 MPa; C_{30}=0.0000244 MPa; α=0.2246026 MPa; β=0.91653;*

Table S17. The model parameters for medium deformation in Table 7

Model	Parameters
Ogden-N3	*α_1=3.1880758; μ_1=0.05834 MPa; α_2=1.495376; μ_2=0.10143955 MPa; α_3=-0.3198387; μ_3=0.198828 MPa;*
Alexander	*C_1=0.13477 MPa; C_2=0.04137 MPa; C_3=0.01422 MPa; k=0.000425; γ=1.17507;*
Yeoh	*C_{10}=0.154369 MPa; C_{20}=0 MPa; C_{30}=0.00001068 MPa;*
Melly	*C_{10}=0.126125 MPa; C_{20}=0.0003406 MPa; C_{30}=0.00001655 MPa; D=0.1538359 MPa;*
Modified Yeoh	*C_{10}=0.15151 MPa; C_{20}=0.000195 MPa; C_{30}=0.00000748 MPa; α=0.0291045 MPa; β=0.5246785;*
Generalized Yeoh	*K_1=0.849356 MPa; m=1; K_2=-0.6802765 MPa; p=1.00653; K_3=0.0000001 MPa; q=4.434723;*
Anssari-Benam	*μ=0.3092459 MPa; N=11.41556785; n=1.0312297;*
Modified Anssari-Benam	*μ=0.217254657 MPa; N=25.766939; n=85; α=0.379018689 MPa; β=0.6344856;*
My work	*C_{10}=0.09435 MPa; C_{20}=0.00141 MPa; C_{30}=0 MPa; α=0.9361 MPa; β=0.35528;*

Table S18. The model parameters for small deformation in Table 7

Model	Parameters
Ogden-N3	*α_1=0.222160; μ_1=0.058955 MPa; α_2=2.632; μ_2=0.1235447 MPa; α_3=0.0444; μ_3=0.176199 MPa;*
Alexander	*C_1=0.129286 MPa; C_2=0.20462658 MPa; C_3=0.00149566 MPa; k=0.0005238; γ=4.23884;*
Yeoh	*C_{10}=0.15199 MPa; C_{20}=0 MPa; C_{30}=2.755E-10 MPa;*
Melly	*C_{10}=0.128929 MPa; C_{20}=0 MPa; C_{30}=0.00001755 MPa; D=0.1580353 MPa;*
Modified Yeoh	*C_{10}=0.00063265 MPa; C_{20}=0.001563569 MPa; C_{30}=0.000005727 MPa; α=0.17600515 MPa; β=0.057149;*
Generalized Yeoh	*K_1=1.07821848 MPa; m=1; K_2=-0.9109487 MPa; p=1.011924; K_3=0.0079585 MPa; q=1.012909;*
Anssari-Benam	*μ=0.3785835 MPa; N=63.874; n=0.168621;*
Modified Anssari-Benam	*μ=0.19769 MPa; N=12.6129; n=1.82063; α=2.03253 MPa; β=0.150266;*
My work	*C_{10}=0.0971 MPa; C_{20}=0.00114 MPa; C_{30}=0 MPa; α=1.90129 MPa; β=0.1717;*

Table S19. The predictive results of different models calibrated by the data from UT and PS of isoprene vulcanized rubber [49].

Models	The number of coefficient	Error	UT	ET	PS
Ogden-N3	6	0.061	0.985	0.847	0.986
Alexander	5	0.068	0.990	0.829	0.977
Yeoh	3	0.095	0.971	0.798	0.947
Melly	4	0.045	0.977	0.935	0.954
ModifiedYeoh	5	0.078	0.981	0.800	0.984
GeneralizedYeoh	6	0.081	0.986	0.791	0.980
Anssari-Benam	3	0.082	0.985	0.787	0.983
Modified Anssari-Benam	5	0.047	0.982	0.903	0.973
**This work**	**5**	**0.041**	**0.974**	**0.911**	**0.993**

Notes: Error indicates the total error of different models; UT, ET and PS represent the goodness of fit of different models for uniaxial tension, equi-biaxial tension and pure shear. "--" indicates that the goodness of fit is negative and the prediction is invalid. The model parameters are shown in Table S23 in supporting information.

Table S20. The predictive results of different models calibrated by the data from UT and PS of unfilled silicone rubber [17].

Models	The number of coefficient	Error	UT	ET	PS
Ogden-N3	6	0.197	0.979	0.460	0.971
Alexander	5	0.323	0.986	0.072	0.974
Yeoh	3	0.523	0.708	--	0.820
Melly	4	0.128	0.980	0.665	0.973
ModifiedYeoh	5	0.117	0.933	0.734	0.981

GeneralizedYeoh	6	0.089	0.931	0.821	0.982
Anssari-Benam	3	0.566	0.937	--	0.973
Modified Anssari-Benam	5	0.666	0.979	--	0.973
**This work**	**5**	**0.134**	**0.982**	**0.642**	**0.974**

Notes: Error indicates the total error of different models; UT, ET and PS represent the goodness of fit of different models for uniaxial tension, equi-biaxial tension and pure shear. "--" indicates that the goodness of fit is negative and the prediction is invalid. The model parameters are shown in Table S24 in supporting information.

Table S21. The predictive results of different models calibrated by the data from UT and PS of poly-arcylamide hydrogel [50].

Models	The number of coefficient	Error	UT	ET	PS
Ogden-N3	6	0.051	0.992	0.864	0.990
Alexander	5	0.057	0.988	0.849	0.991
Yeoh	3	0.091	0.971	0.771	0.985
Melly	4	0.043	0.989	0.891	0.990
ModifiedYeoh	5	0.090	0.973	0.768	0.990
GeneralizedYeoh	6	0.091	0.974	0.764	0.990
Anssari-Benam	3	0.097	0.954	0.770	0.986
Modified Anssari-Benam	5	0.043	0.990	0.890	0.991
**This work**	**5**	**0.035**	**0.992**	**0.914**	**0.990**

Notes: Error indicates the total error of different models; UT, ET and PS represent the goodness of fit of different models for uniaxial tension, equi-biaxial tension and pure shear. "--" indicates that the goodness of fit is negative and the prediction is invalid. The model parameters are shown in Table S25 in supporting information.

Table S22. The predictive results of different models calibrated by the data from UT and PS of carbon-black-filled styrene butadiene rubber [51].

Models	The number of coefficient	Error	UT	ET	PS
Ogden-N3	6	0.041	0.968	0.952	0.957
Alexander	5	0.037	0.981	0.927	0.982
Yeoh	3	0.126	0.925	0.790	0.907
Melly	4	0.066	0.963	0.901	0.937
ModifiedYeoh	5	0.113	0.967	0.767	0.927
GeneralizedYeoh	6	0.109	0.968	0.776	0.927
Anssari-Benam	3	0.157	0.942	0.660	0.925
Modified Anssari-Benam	5	0.046	0.966	0.940	0.957
**This work**	**5**	**0.049**	**0.968**	**0.938**	**0.949**

Notes: Error indicates the total error of different models; UT, ET and PS represent the goodness of fit of different models for uniaxial tension, equi-biaxial tension and pure shear. "--" indicates that the goodness of fit is negative and the prediction is invalid. The model parameters are shown in Table S26 in supporting information.

Table S23. The parameters of different model calibrated by the data from UT and PS of isoprene vulcanized rubber

Model	Parameters
Ogden-N3	$\alpha_1=9.72856$; $\mu_1=0.0000012$ MPa; $\alpha_2=4.88164$; $\mu_2=0.00112$ MPa; $\alpha_3=1.58782$; $\mu_3=0.38452$ MPa;
Alexander	$C_1=0.14810$ MPa; $C_2=0.08285$ MPa; $C_3=0$ MPa; $k=0$; $\gamma=1.2627859$;
Yeoh	$C_{10}=0.155407$ MPa; $C_{20}=1E-8$ MPa; $C_{30}=0$ MPa;
Melly	$C_{10}=0.1491316$ MPa; $C_{20}=0$ MPa; $C_{30}=0$ MPa; $D=0.06775$ MPa;
Modified Yeoh	$C_{10}=0.12979$ MPa; $C_{20}=0.000289$ MPa; $C_{30}=0.00001556$ MPa; $\alpha=0.059919$ MPa; $\beta=0.161331$;
Generalized Yeoh	$K_1=0.2295233$ MPa; $m=1$; $K_2=-0.065447$ MPa; $p=1.397285937$; $K_3=0.026765$ MPa; $q=1.5599$;
Anssari-Benam	$\mu=0.081174$ MPa; $N=0.357115$; $n=0.28277$;
Modified Anssari-Benam	$\mu=0.719328$ MPa; $N=0.0000026$; $n=2.6356154$; $\alpha=6.55401847$ MPa; $\beta=0.0289229$;
My work	$C_{10}=1.376388$ MPa; $C_{20}=0.00104$ MPa; $C_{30}=-0.0000041$ MPa; $\alpha=2.29319$ MPa; $\beta=-2.064195$;

Table S24. The parameters of different model calibrated by the data from UT and PS of unfilled silicone rubber

Model	Parameters
Ogden-N3	$\alpha_1=1.11700$; $\mu_1=0.308509$ MPa; $\alpha_2=8.022885$; $\mu_2=0.00587$ MPa; $\alpha_3=-4.23368$; $\mu_3=0.013435$ MPa;
Alexander	$C_1=0.126247$ MPa; $C_2=0.09678$ MPa; $C_3=0.00278596$ MPa; $k=0.05734279$; $\gamma=2.2575024$;
Yeoh	$C_{10}=0.1206157$ MPa; $C_{20}=0$ MPa; $C_{30}=0.0158067$ MPa;
Melly	$C_{10}=0.127741686$ MPa; $C_{20}=0$ MPa; $C_{30}=0.002757$ MPa; $D=0.12116189$ MPa;
Modified Yeoh	$C_{10}=0.1298157$ MPa; $C_{20}=0.000001266$ MPa; $C_{30}=0.00327815$ MPa; $\alpha=0.057514488$ MPa; $\beta=0.681829$;

Generalized Yeoh	K_1=0.20068558 MPa; m=1; K_2=-0.1 MPa; p=1.973845; K_3=0.075507 MPa; q=2.17729;
Anssari-Benam	μ=0.318809 MPa; N=2.1757257; n=1.0477485;
Modified Anssari-Benam	μ=0.1794 MPa; N=2.4882355; n=1.4125138; α=0.822476 MPa; β=0.270858;
My work	C_{10}=0.101608 MPa; C_{20}=0 MPa; C_{30}=0.0030489 MPa; α=1.484448 MPa; β=0.1724878;

Table S25. The parameters of different model calibrated by the data from UT and PS of poly-arcylamide hydrogel

**Model**	**Parameters**
Ogden-N3	α_1=1.80246; μ_1=0.00231 MPa; α_2=0.025249; μ_2=0.0026228 MPa; α_3=3.34351; μ_3=0.000819 MPa;
Alexander	C_1=0.002433 MPa; C_2=0.0027534 MPa; C_3=0.0000001639 MPa; k=0.0009197; γ=7.36328;
Yeoh	C_{10}=0.00268 MPa; C_{20}=0 MPa; C_{30}=3.725E-7 MPa;
Melly	C_{10}=0.00244 MPa; C_{20}=1.34E-7 MPa; C_{30}=7.7486E-7 MPa; D=0.00122 MPa;
Modified Yeoh	C_{10}=0.00221165 MPa; C_{20}=0.000023585 MPa; C_{30}=0 MPa; α=0.000607 MPa; β=0.1799296;
Generalized Yeoh	K_1=0.92755 MPa; m=1; K_2=-0.924766 MPa; p=1.07449; K_3=0.0000001 MPa; q=3.6293;
Anssari-Benam	μ=0.066565 MPa; N=0.0036; n=11.987967;
Modified Anssari-Benam	μ=0.0039 MPa; N=18.9523; n=32.1626; α=0.024685 MPa; β=0.12702178;
My work	C_{10}=0.0018258 MPa; C_{20}=0.0000306 MPa; C_{30}=0 MPa; α=1.7097385 MPa; β=0.002398;

Table S26. The parameters of different model calibrated by the data from UT and PS of carbon-black-filled styrene butadiene rubber

**Model**	**Parameters**
Ogden-N3	α_1=0.75172; μ_1=0.8629886 MPa; α_2=0.7517155; μ_2=0.59426 MPa; α_3=0.751706; μ_3=0.09592 MPa;
Alexander	C_1=0.488956 MPa; C_2=0.04274595 MPa; C_3=0.1425747 MPa; k=5.2357E-8; γ=0.0801276;
Yeoh	C_{10}=0.64822856 MPa; C_{20}=0 MPa; C_{30}=0 MPa;
Melly	C_{10}=0.465135 MPa; C_{20}=0 MPa; C_{30}=0 MPa; D=0.9250067 MPa;
Modified Yeoh	C_{10}=0.62163597 MPa; C_{20}=2.23E-8 MPa; C_{30}=0 MPa; α=0.344689 MPa; β=4.89252;
Generalized Yeoh	K_1=1.78476899 MPa; m=1; K_2=-1.32311778 MPa; p=1.15276; K_3=0.23359 MPa; q=1.555475;
Anssari-Benam	μ=2.0111 MPa; N=51.836617; n=0.067696;
Modified Anssari-Benam	μ=0.87155 MPa; N=13.5647; n=0.274455; α=16.056696 MPa; β=0.10759;
My work	C_{10}=0.26596 MPa; C_{20}=3.159E-6 MPa; C_{30}=0 MPa; α=16.17489 MPa; β=0.12210187;

Table S27 The parameters of the constitutive model corresponding to the Table 9.

Methods	Arruda-Boyce model	Ogden-3N model	Yeoh model
ABAQUS	μ=0.3053 MPa, λ_m=5.1322 MPa	α_1=1.3913 MPa; μ_1=0.3430 MPa;<br>α_2=4.7869 MPa; μ_2=0.0044 MPa;<br>α_3=-1.6503 MPa; μ_3=0.0240 MPa;	C_{10}=0.1712 MPa<br>C_{20}=-0.0008 MPa<br>C_{30}=0.00003 MPa
This work	μ=0.2797 MPa, λ_m=4.7589 MPa	α_1=4.7203 MPa; μ_1=0.0053 MPa;<br>α_2=1.3336 MPa; μ_2=0.3572 MPa;<br>α_3=-1.8124 MPa; μ_3=0.0163 MPa;	C_{10}=0.1631 MPa<br>C_{20}=-0.0006 MPa<br>C_{30}=0.00003 MPa

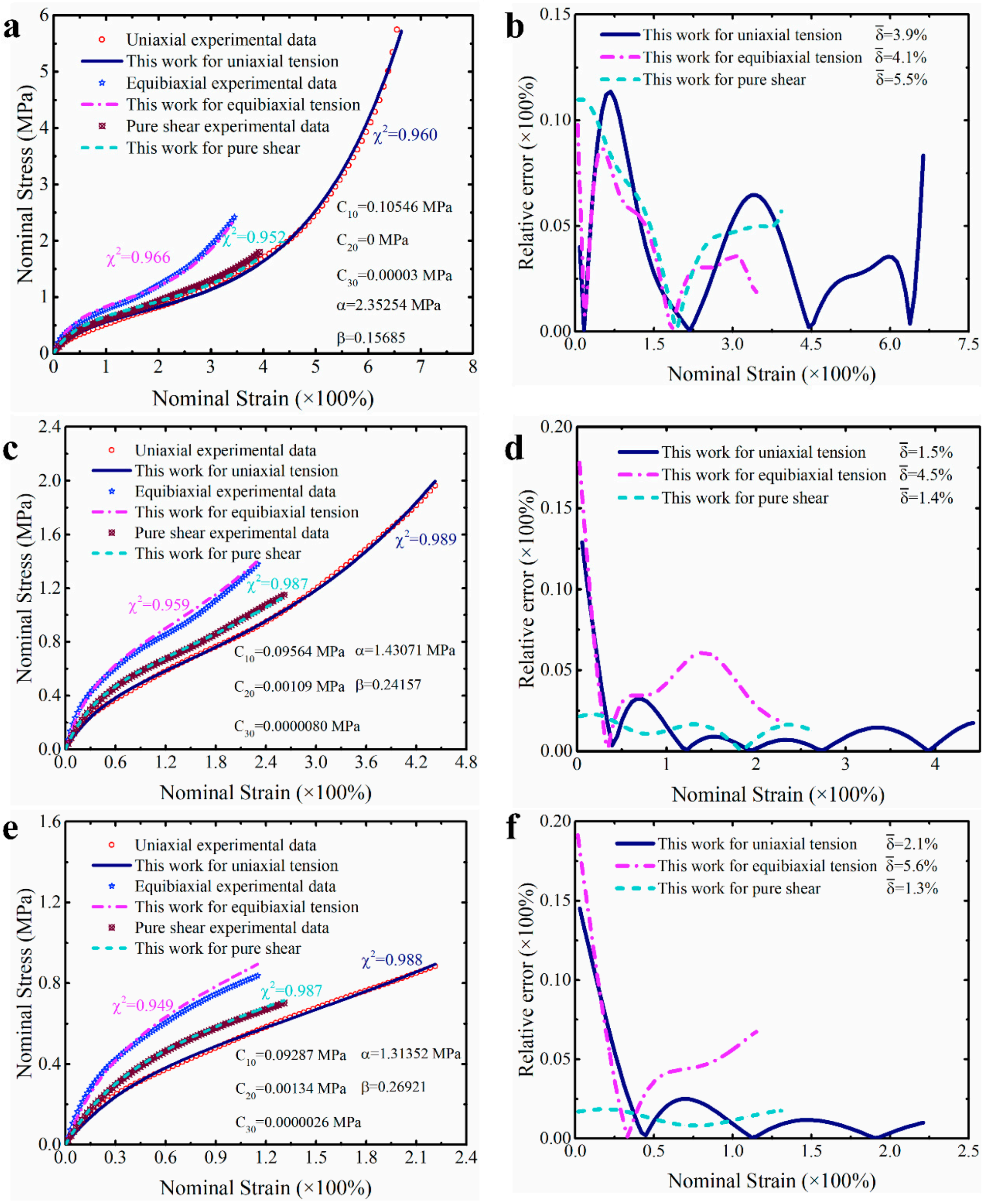

Figure S1. The prediction effects of the modified model for different deformation modes with different deformation ranges. (a-b) in large deformation, (c-d) in medium deformation, (e-f) in small deformation. Here, the parameters of the modified model are calibrated only by the Treloar's uniaxial experimental data with different deformation ranges.

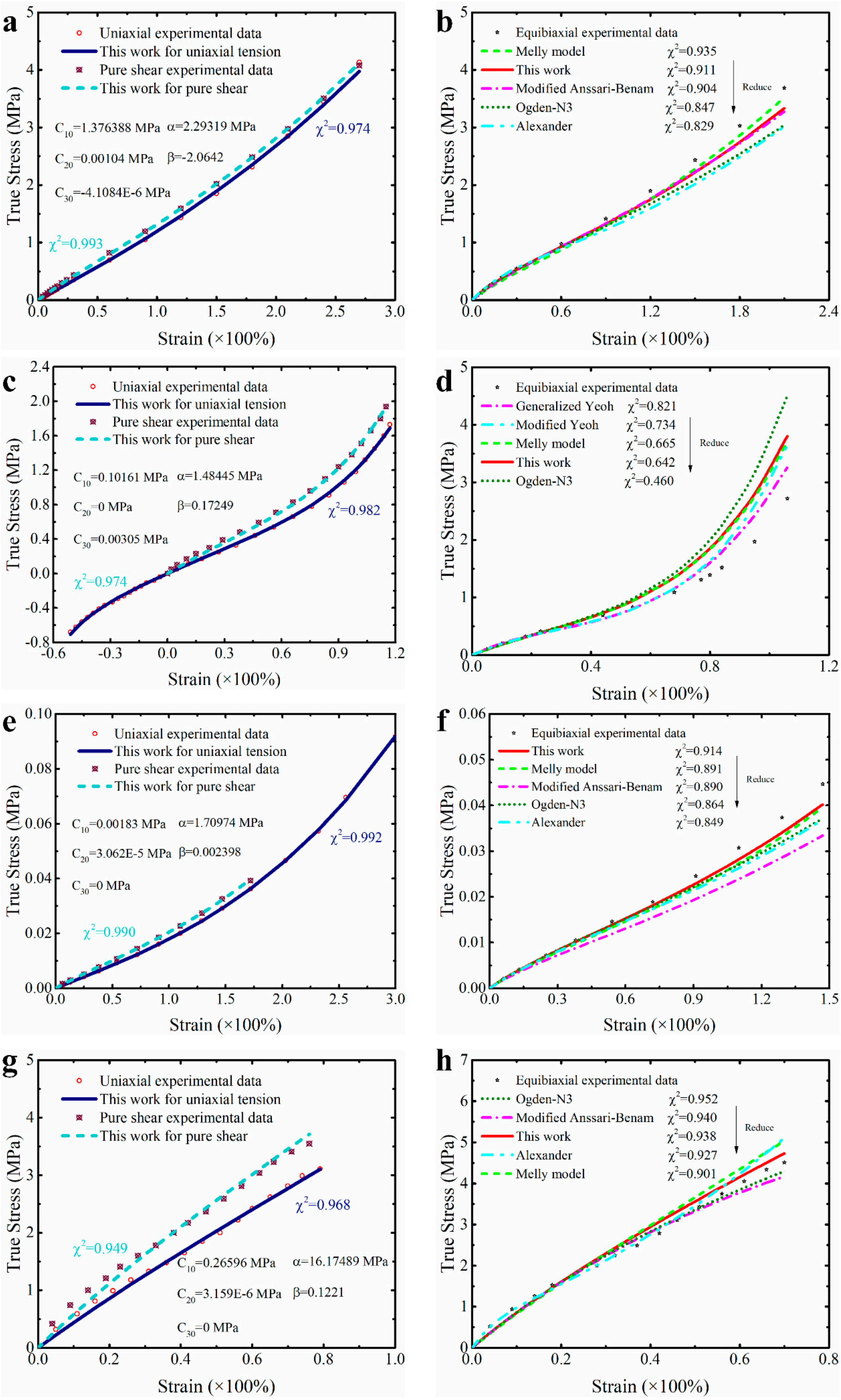

Figure S2. The prediction effects of the modified model for different deformation modes of different materials in the case where the parameters of model are calibrated by the data only from UT and PS. (a-b) Isoprene vulcanized rubber. (c-d) Unfilled silicone rubber. (e-f)

Poly-acrylamide hydrogel. (g-h) Carbon-black-filled styrene butadiene rubber. Only the top five constitutive models are listed on the right side of the figure.

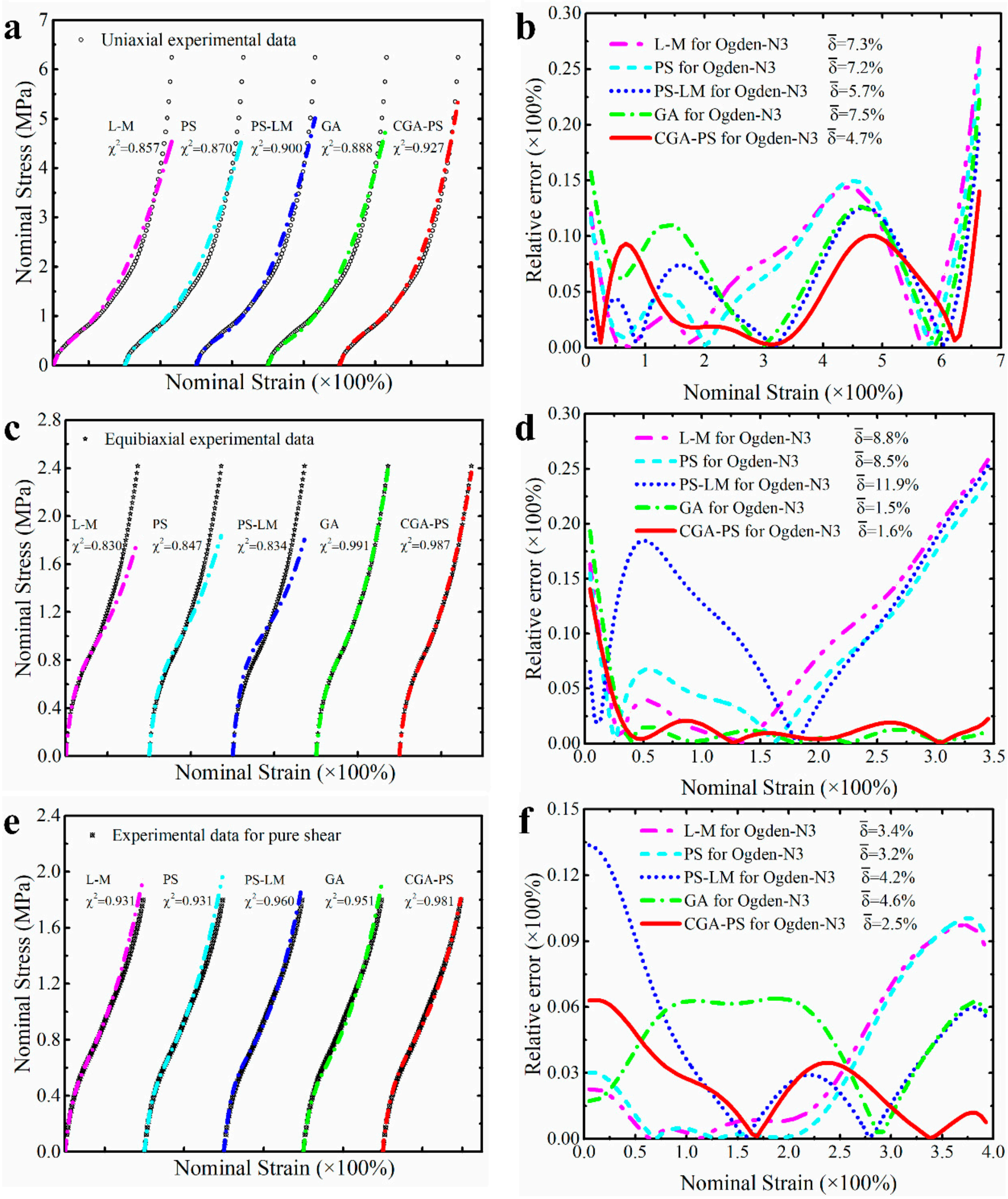

Figure S3. The comparison of prediction performance by the third-order Ogden model based on different methods of parameter identification. (a-b) the prediction performance of uniaxial tension by the modified model based on different methods of parameter identification. (c-d) the prediction performance of equi-biaxial tension by the modified model based on different methods of parameter identification. (e-f) the prediction performance of pure shear by the modified model based on different methods of parameter identification. $\overline{\delta}$ means the average relative error.

Equation S1. Components of the Hessian matrix ***H***

$$
\begin{aligned}
H_{11} &= C_{10}\cdot\left(2+6\lambda_1^{-4}\lambda_2^{-2}\right)+2C_{20}\cdot\left(2\lambda_1-2\lambda_1^{-3}\cdot\lambda_2^{-2}\right)\cdot\left(2\lambda_1-2\lambda_1^{-3}\cdot\lambda_2^{-2}\right)\\
&+2C_{20}\cdot\left(\lambda_1^{2}+\lambda_2^{2}+\lambda_1^{-2}\cdot\lambda_2^{-2}-3\right)\cdot\left(2+6\lambda_1^{-4}\cdot\lambda_2^{-2}\right)\\
&+6C_{30}\cdot\left(\lambda_1^{2}+\lambda_2^{2}+\lambda_1^{-2}\cdot\lambda_2^{-2}-3\right)\cdot\left(2\lambda_1-2\lambda_1^{-3}\cdot\lambda_2^{-2}\right)\cdot\left(2\lambda_1-2\lambda_1^{-3}\cdot\lambda_2^{-2}\right)\\
&+3C_{30}\cdot\left(\lambda_1^{2}+\lambda_2^{2}+\lambda_1^{-2}\cdot\lambda_2^{-2}-3\right)^{2}\cdot\left(2+6\lambda_1^{-4}\cdot\lambda_2^{-2}\right)\\
&+\alpha\cdot\left[(\beta-1)\lambda_1^{\beta-2}\cdot\lambda_2^{\beta}+(\beta+1)\lambda_1^{-\beta-2}\right]\\
H_{22} &= C_{10}\cdot\left(2+6\lambda_1^{-2}\lambda_2^{-4}\right)+2C_{20}\cdot\left(2\lambda_2-2\lambda_1^{-2}\cdot\lambda_2^{-3}\right)\cdot\left(2\lambda_2-2\lambda_1^{-2}\cdot\lambda_2^{-3}\right)\\
&+2C_{20}\cdot\left(\lambda_1^{2}+\lambda_2^{2}+\lambda_1^{-2}\cdot\lambda_2^{-2}-3\right)\cdot\left(2+6\lambda_1^{-2}\cdot\lambda_2^{-4}\right)\\
&+6C_{30}\cdot\left(\lambda_1^{2}+\lambda_2^{2}+\lambda_1^{-2}\cdot\lambda_2^{-2}-3\right)\cdot\left(2\lambda_2-2\lambda_1^{-2}\cdot\lambda_2^{-3}\right)\cdot\left(2\lambda_2-2\lambda_1^{-2}\cdot\lambda_2^{-3}\right)\\
&+3C_{30}\cdot\left(\lambda_1^{2}+\lambda_2^{2}+\lambda_1^{-2}\cdot\lambda_2^{-2}-3\right)^{2}\cdot\left(2+6\lambda_1^{-2}\cdot\lambda_2^{-4}\right)\\
&+\alpha\cdot\left[(\beta-1)\lambda_1^{\beta}\cdot\lambda_2^{\beta-2}+(\beta+1)\lambda_2^{-\beta-2}\right]\\
H_{12} = H_{21} &= C_{10}\cdot\left(4\lambda_1^{-3}\lambda_2^{-3}\right)+2C_{20}\cdot\left(2\lambda_2-2\lambda_1^{-2}\cdot\lambda_2^{-3}\right)\cdot\left(2\lambda_1-2\lambda_1^{-3}\cdot\lambda_2^{-2}\right)\\
&+2C_{20}\cdot\left(\lambda_1^{2}+\lambda_2^{2}+\lambda_1^{-2}\cdot\lambda_2^{-2}-3\right)\cdot\left(4\lambda_1^{-3}\cdot\lambda_2^{-3}\right)\\
&+6C_{30}\cdot\left(\lambda_1^{2}+\lambda_2^{2}+\lambda_1^{-2}\cdot\lambda_2^{-2}-3\right)\cdot\left(2\lambda_2-2\lambda_1^{-2}\cdot\lambda_2^{-3}\right)\cdot\left(2\lambda_1-2\lambda_1^{-3}\cdot\lambda_2^{-2}\right)\\
&+3C_{30}\cdot\left(\lambda_1^{2}+\lambda_2^{2}+\lambda_1^{-2}\cdot\lambda_2^{-2}-3\right)^{2}\cdot\left(4\lambda_1^{-3}\cdot\lambda_2^{-3}\right)\\
&+\alpha\cdot\left(\beta\lambda_1^{\beta-1}\cdot\lambda_2^{\beta-1}\right)
\end{aligned}
$$

Equation S2. Components of the matrix ***D***

$$
\begin{aligned}
D_{11} &= \Big[C_{10}\cdot\left(4\lambda_1+4\lambda_1^{-3}\lambda_2^{-2}\right)+2C_{20}\cdot\left(2\lambda_1-2\lambda_1^{-3}\cdot\lambda_2^{-2}\right)\cdot\left(2\lambda_1^{2}-2\lambda_1^{-2}\cdot\lambda_2^{-2}\right)\\
&+2C_{20}\cdot\left(\lambda_1^{2}+\lambda_2^{2}+\lambda_1^{-2}\cdot\lambda_2^{-2}-3\right)\cdot\left(4\lambda_1+4\lambda_1^{-3}\cdot\lambda_2^{-2}\right)\\
&+3C_{30}\cdot\left(\lambda_1^{2}+\lambda_2^{2}+\lambda_1^{-2}\cdot\lambda_2^{-2}-3\right)^{2}\cdot\left(4\lambda_1+4\lambda_1^{-3}\cdot\lambda_2^{-2}\right)\\
&+6C_{30}\cdot\left(\lambda_1^{2}+\lambda_2^{2}+\lambda_1^{-2}\cdot\lambda_2^{-2}-3\right)\cdot\left(2\lambda_1-2\lambda_1^{-3}\cdot\lambda_2^{-2}\right)\cdot\left(2\lambda_1^{2}-2\lambda_1^{-2}\cdot\lambda_2^{-2}\right)\\
&+\alpha\cdot\left(\beta\lambda_1^{\beta-1}\cdot\lambda_2^{\beta}+\beta\lambda_1^{-\beta-1}\right)\Big]\bullet\lambda_1
\end{aligned}
$$

$$
\begin{aligned}
D_{22} &= \Big[C_{10}\cdot\left(4\lambda_2+4\lambda_1^{-2}\lambda_2^{-3}\right)+2C_{20}\cdot\left(2\lambda_2-2\lambda_1^{-2}\cdot\lambda_2^{-3}\right)\cdot\left(2\lambda_2^{2}-2\lambda_1^{-2}\cdot\lambda_2^{-2}\right)\\
&+2C_{20}\cdot\left(\lambda_1^{2}+\lambda_2^{2}+\lambda_1^{-2}\cdot\lambda_2^{-2}-3\right)\cdot\left(4\lambda_2+4\lambda_1^{-2}\cdot\lambda_2^{-3}\right)\\
&+3C_{30}\cdot\left(\lambda_1^{2}+\lambda_2^{2}+\lambda_1^{-2}\cdot\lambda_2^{-2}-3\right)^{2}\cdot\left(4\lambda_2+4\lambda_1^{-2}\cdot\lambda_2^{-3}\right)\\
&+6C_{30}\cdot\left(\lambda_1^{2}+\lambda_2^{2}+\lambda_1^{-2}\cdot\lambda_2^{-2}-3\right)\cdot\left(2\lambda_2-2\lambda_1^{-2}\cdot\lambda_2^{-3}\right)\cdot\left(2\lambda_2^{2}-2\lambda_1^{-2}\cdot\lambda_2^{-2}\right)\\
&+\alpha\cdot\left(\beta\lambda_1^{\beta}\cdot\lambda_2^{\beta-1}+\beta\lambda_2^{-\beta-1}\right)\Big]\bullet\lambda_2
\end{aligned}
$$

$$
\begin{aligned}
D_{12} = D_{21} &= C_{10}\cdot 4\lambda_1^{-2}\lambda_2^{-2} + 2C_{20}\cdot\left(2\lambda_2^{\,2} - 2\lambda_1^{-2}\cdot\lambda_2^{-2}\right)\cdot\left(2\lambda_1^{\,2} - 2\lambda_1^{-2}\cdot\lambda_2^{-2}\right)\\
&+2C_{20}\cdot\left(\lambda_1^{\,2} + \lambda_2^{\,2} + \lambda_1^{-2}\cdot\lambda_2^{-2} - 3\right)\cdot\left(4\lambda_1^{-2}\cdot\lambda_2^{-2}\right)\\
&+3C_{30}\cdot\left(\lambda_1^{\,2} + \lambda_2^{\,2} + \lambda_1^{-2}\cdot\lambda_2^{-2} - 3\right)^2\cdot\left(4\lambda_1^{-2}\cdot\lambda_2^{-2}\right)\\
&+6C_{30}\cdot\left(\lambda_1^{\,2} + \lambda_2^{\,2} + \lambda_1^{-2}\cdot\lambda_2^{-2} - 3\right)\cdot\left(2\lambda_2^{\,2} - 2\lambda_1^{-2}\cdot\lambda_2^{-2}\right)\cdot\left(2\lambda_1^{\,2} - 2\lambda_1^{-2}\cdot\lambda_2^{-2}\right)\\
&+\alpha\cdot\left(\beta\lambda_1^{\beta}\cdot\lambda_2^{\beta}\right)
\end{aligned}
$$

Equation S3. Some constitutive models for comparison

1）Third-order Ogden model

$$W = \sum_{i=1}^{3}\frac{2\mu_i}{\alpha_i^2}\left(\lambda_1^{\alpha_i} + \lambda_2^{\alpha_i} + \lambda_3^{\alpha_i} - 3\right)$$

$$T_{UT} = \sum_{i=1}^{n}\frac{2\mu_i}{\alpha_i}\left(\lambda^{\alpha_i - 1} - \lambda^{-\frac{\alpha_i}{2} - 1}\right)$$

$$T_{ET} = \sum_{i=1}^{n}\frac{2\mu_i}{\alpha_i}\left(\lambda^{\alpha_i - 1} - \lambda^{-2\alpha_i - 1}\right)$$

$$T_{PS} = \sum_{i=1}^{n}\frac{2\mu_i}{\alpha_i}\left(\lambda^{\alpha_i - 1} - \lambda^{-\alpha_i - 1}\right)$$

2）Alexander model

$$W = C_1\int \exp\left[k\left(\mathrm{I}_1 - 3\right)^2\right] d\mathrm{I}_1 + C_2\ln\left[\frac{\left(\mathrm{I}_2 - 3\right) + \gamma}{\gamma}\right] + C_3\left(\mathrm{I}_2 - 3\right)$$

$$T_{UT} = 2\bullet C_1\bullet e^{\left[k(I_1-3)^2\right]}\bullet\left(\lambda - \frac{1}{\lambda^2}\right) + 2\bullet\left[\frac{C_2}{\left(I_2 - 3\right) + \gamma} + C_3\right]\bullet\left(1 - \frac{1}{\lambda^3}\right)$$

$$T_{ET} = 2\bullet C_1\bullet e^{\left[k(I_1-3)^2\right]}\bullet\left(\lambda - \frac{1}{\lambda^5}\right) + 2\bullet\left[\frac{C_2}{\left(I_2 - 3\right) + \gamma} + C_3\right]\bullet\left(\lambda^3 - \frac{1}{\lambda^3}\right)$$

$$T_{PS} = 2\bullet C_1\bullet e^{\left[k(I_1-3)^2\right]}\bullet\left(\lambda - \frac{1}{\lambda^3}\right) + 2\bullet\left[\frac{C_2}{\left(I_2 - 3\right) + \gamma} + C_3\right]\bullet\left(\lambda - \frac{1}{\lambda^3}\right)$$

3）Yeoh model

$$W = C_{10}\left(\mathrm{I}_1 - 3\right) + C_{20}\left(\mathrm{I}_1 - 3\right)^2 + C_{30}\left(\mathrm{I}_1 - 3\right)^3$$

$$T_{UT} = 2\bullet\left[C_{10} + 2C_{20}\left(\lambda^2 + \frac{2}{\lambda} - 3\right) + 3C_{30}\left(\lambda^2 + \frac{2}{\lambda} - 3\right)^2\right]\left(\lambda - \frac{1}{\lambda^2}\right)$$

$$T_{ET} = 2\bullet\left[C_{10} + 2C_{20}\left(2\lambda^2 + \frac{1}{\lambda^4} - 3\right) + 3C_{30}\left(2\lambda^2 + \frac{1}{\lambda^4} - 3\right)^2\right]\left(\lambda - \frac{1}{\lambda^5}\right)$$

$$T_{PS} = 2\bullet\left[C_{10} + 2C_{20}\left(\lambda^2 + \frac{1}{\lambda^2} - 2\right) + 3C_{30}\left(\lambda^2 + \frac{1}{\lambda^2} - 2\right)^2\right]\left(\lambda - \frac{1}{\lambda^3}\right)$$

4) Melly model

$$W = C_{10}\left(\mathrm{I}_1 - 3\right) + C_{20}\left(\mathrm{I}_1 - 3\right)^2 + C_{30}\left(\mathrm{I}_1 - 3\right)^3 + D\left(\sqrt{\mathrm{I}_2} - \sqrt{3}\right)$$

$$T_{UT} = 2\bullet\left[C_{10} + 2C_{20}\left(\lambda^2 + \frac{2}{\lambda} - 3\right) + 3C_{30}\left(\lambda^2 + \frac{2}{\lambda} - 3\right)^2\right]\left(\lambda - \frac{1}{\lambda^2}\right) + D\bullet\left(\frac{1}{\lambda^2} + 2\lambda\right)^{-\frac{1}{2}}\bullet\left(1 - \frac{1}{\lambda^3}\right)$$

$$T_{ET} = 2\bullet\left[C_{10} + 2C_{20}\left(2\lambda^2 + \frac{1}{\lambda^4} - 3\right) + 3C_{30}\left(2\lambda^2 + \frac{1}{\lambda^4} - 3\right)^2\right]\left(\lambda - \frac{1}{\lambda^5}\right) + D\bullet\left(\frac{2}{\lambda^2} + \lambda^4\right)^{-\frac{1}{2}}\bullet\left(\lambda^3 - \frac{1}{\lambda^3}\right)$$

$$T_{PS} = 2\bullet\left[C_{10} + 2C_{20}\left(\lambda^2 + \frac{1}{\lambda^2} - 2\right) + 3C_{30}\left(\lambda^2 + \frac{1}{\lambda^2} - 2\right)^2\right]\left(\lambda - \frac{1}{\lambda^3}\right) + D\bullet\left(\frac{1}{\lambda^2} + \lambda^2 + 1\right)^{-\frac{1}{2}}\bullet\left(\lambda - \frac{1}{\lambda^3}\right)$$

5) Modified Yeoh model

$$W = C_{10}\left(\mathrm{I}_1 - 3\right) + C_{20}\left(\mathrm{I}_1 - 3\right)^2 + C_{30}\left(\mathrm{I}_1 - 3\right)^3 + \frac{\alpha}{\beta}\left(1 - e^{-\beta(\mathrm{I}_1 - 3)}\right)$$

$$T_{UT} = 2\bullet\left[C_{10} + 2C_{20}\left(\lambda^2 + \frac{2}{\lambda} - 3\right) + 3C_{30}\left(\lambda^2 + \frac{2}{\lambda} - 3\right)^2 + \alpha\bullet e^{-\beta\left(\lambda^2 + \frac{2}{\lambda} - 3\right)}\right]\left(\lambda - \frac{1}{\lambda^2}\right)$$

$$T_{ET} = 2\bullet\left[C_{10} + 2C_{20}\left(2\lambda^2 + \frac{1}{\lambda^4} - 3\right) + 3C_{30}\left(2\lambda^2 + \frac{1}{\lambda^4} - 3\right)^2 + \alpha\bullet e^{-\beta\left(2\lambda^2 + \frac{1}{\lambda^4} - 3\right)}\right]\left(\lambda - \frac{1}{\lambda^5}\right)$$

$$T_{PS} = 2\bullet\left[C_{10} + 2C_{20}\left(\lambda^2 + \frac{1}{\lambda^2} - 2\right) + 3C_{30}\left(\lambda^2 + \frac{1}{\lambda^2} - 2\right)^2 + \alpha\bullet e^{-\beta\left(\lambda^2 + \frac{1}{\lambda^2} - 2\right)}\right]\left(\lambda - \frac{1}{\lambda^3}\right)$$

6) Generalized Yeoh model

$$W = K_1\left(\mathrm{I}_1 - 3\right)^m + K_2\left(\mathrm{I}_1 - 3\right)^p + K_3\left(\mathrm{I}_1 - 3\right)^q$$

$$T_{UT} = 2\bullet\left(\lambda - \frac{1}{\lambda^2}\right)\bullet\left[K_1\bullet m\bullet\left(\lambda^2 + \frac{2}{\lambda} - 3\right)^{m-1} + K_2\bullet p\bullet\left(\lambda^2 + \frac{2}{\lambda} - 3\right)^{p-1} + K_3\bullet q\bullet\left(\lambda^2 + \frac{2}{\lambda} - 3\right)^{q-1}\right]$$

$$T_{ET} = 2\bullet\left(\lambda - \frac{1}{\lambda^5}\right)\bullet\left[K_1\bullet m\bullet\left(2\lambda^2 + \frac{1}{\lambda^4} - 3\right)^{m-1} + K_2\bullet p\bullet\left(2\lambda^2 + \frac{1}{\lambda^4} - 3\right)^{p-1} + K_3\bullet q\bullet\left(2\lambda^2 + \frac{1}{\lambda^4} - 3\right)^{q-1}\right]$$

$$T_{PS} = 2\bullet\left(\lambda - \frac{1}{\lambda^3}\right)\bullet\left[K_1\bullet m\bullet\left(\lambda^2 + \frac{1}{\lambda^2} - 2\right)^{m-1} + K_2\bullet p\bullet\left(\lambda^2 + \frac{1}{\lambda^2} - 2\right)^{p-1} + K_3\bullet q\bullet\left(\lambda^2 + \frac{1}{\lambda^2} - 2\right)^{q-1}\right]$$

7) Anssari-Benam model

$$W = \frac{3(n-1)}{2n}\mu N\left[\frac{1}{3N(n-1)}\left(\mathrm{I}_1 - 3\right) - \ln\left(\frac{\mathrm{I}_1 - 3N}{3 - 3N}\right)\right]$$

$$T_{UT} = \frac{1}{\lambda}\bullet\left\{\frac{\mu}{n}\left(\frac{\lambda^2 + 2\lambda^{-1} - 3nN}{\lambda^2 + 2\lambda^{-1} - 3N}\right)\left(\lambda^2 - \frac{1}{\lambda}\right)\right\}$$

$$T_{ET} = \frac{1}{\lambda} \bullet \left\{ \frac{\mu}{n} \left(\frac{2\lambda^2 + \lambda^{-4} - 3nN}{2\lambda^2 + \lambda^{-4} - 3N} \right) \left(\lambda^2 - \frac{1}{\lambda^4} \right) \right\}$$

$$T_{PS} = \frac{1}{\lambda} \bullet \left\{ \frac{\mu}{n} \left(\frac{\lambda^2 + \lambda^{-2} + 1 - 3nN}{\lambda^2 + \lambda^{-2} + 1 - 3N} \right) \left(\lambda^2 - \frac{1}{\lambda^2} \right) \right\}$$

8) Modified Anssari-Benam model

$$W = \frac{3(n-1)}{2n} \mu N \left[\frac{1}{3N(n-1)} (\mathrm{I}_1 - 3) - \ln \left(\frac{\mathrm{I}_1 - 3N}{3 - 3N} \right) \right] + \frac{\alpha}{\beta} \left[(\lambda_1 \lambda_2)^{\beta} + (\lambda_2 \lambda_3)^{\beta} + (\lambda_1 \lambda_3)^{\beta} - 3 \right]$$

$$T_{UT} = \frac{1}{\lambda} \bullet \left\{ \frac{\mu}{n} \left(\frac{\lambda^2 + 2\lambda^{-1} - 3nN}{\lambda^2 + 2\lambda^{-1} - 3N} \right) \left(\lambda^2 - \frac{1}{\lambda} \right) + \alpha \bullet \left(\lambda^{\frac{\beta}{2}} - \lambda^{-\beta} \right) \right\}$$

$$T_{ET} = \frac{1}{\lambda} \bullet \left\{ \frac{\mu}{n} \left(\frac{2\lambda^2 + \lambda^{-4} - 3nN}{2\lambda^2 + \lambda^{-4} - 3N} \right) \left(\lambda^2 - \frac{1}{\lambda^4} \right) + \alpha \bullet \left(\lambda^{2\beta} - \lambda^{-\beta} \right) \right\}$$

$$T_{PS} = \frac{1}{\lambda} \bullet \left\{ \frac{\mu}{n} \left(\frac{\lambda^2 + \lambda^{-2} + 1 - 3nN}{\lambda^2 + \lambda^{-2} + 1 - 3N} \right) \left(\lambda^2 - \frac{1}{\lambda^2} \right) + \alpha \bullet \left(\lambda^{\beta} - \lambda^{-\beta} \right) \right\}$$
